# Conformational Preferences for N‑Glycans at the Surface of CEACAM1-Ig1

**DOI:** 10.1021/acschembio.5c00746

**Published:** 2025-12-19

**Authors:** Alexander Eletsky, Chin Huang, Yinglong Miao, Kelley W. Moremen, Laura C. Morris, James H. Prestegard

**Affiliations:** † Complex Carbohydrate Research Center, 1355 University of Georgia, Athens, Georgia 30602, United States; ‡ Department of Pharmacology and Computational Medicine Program, 2331 University of North Carolina−Chapel Hill, Chapel Hill, North Carolina 27599, United States

## Abstract

Glycans on glycoproteins play roles that range from quality control in protein folding, to mediation of interactions with other proteins, to stabilization of the protein to which they are attached. Computation can suggest structures that underlie these roles, but confidence is limited by the accuracy of energetic calculations and their applicability to the aqueous environment in which proteins function. Experimental validation of suggested structures is therefore of primary importance. Here we use NMR data, including long-range pseudocontact shifts (PCSs) and residual dipolar couplings (RDCs), to screen structures produced by a version of accelerated molecular dynamics (Pep-GaMD). This version was designed to improve the search for peptide–protein interactions, but here it is successfully applied to glycans attached to a target protein. The target protein, the N-terminal domain of human CEACAM1, is expressed with homogeneous GlcNAc_2_Man_5_ glycans at its three N-glycosylation sites. One site (N104) is found to have preferred conformations that exploit hydrophobic interactions between its glycans and protein hydrophobic residues, potentially adding to protein stability and protection from adverse interactions.

## Graphical abstract

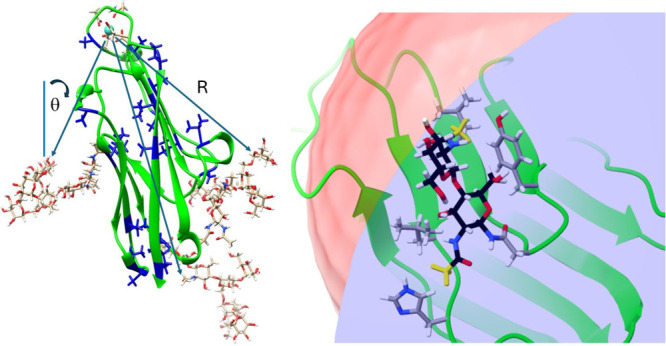

## Introduction

N-glycosylation is a common post/cotranslational modification of eukaryotic proteins that begins in the endoplasmic reticulum (ER) with the transfer of an extended glycan structure comprised of three glucose residues, nine mannose residues and two N-acetylglucosamine residues to the asparagine of a three residue sequon (Asn-X-Ser/Thr).^1^ The role of this addition in assuring proper protein folding is well recognized,^2^ as is the role of fully elaborated N-glycans in mediating a host of glycan-protein interactions.^3^ Other than attachment to the surface of a glycoprotein, it is not clear that more specific interactions between the glycan and its own protein surface would be required for these roles. Also, glycans are often depicted as being highly dynamic, sampling many conformations that extend well away from the protein surface.^4,5^ Despite being suggested long ago,^6^ the extent to which additional nonbonded glycan-protein interactions occur and the extent to which they play roles in protein stability, or protection of exposed surface sites from undesirable pathogenic interactions, remains a question. Recent computational studies have emphasized some specific interactions between attached glycans and aromatic groups two residues prior to the attachment site in what is termed an ‘enhanced aromatic sequon’. Both C–H-π and hydrophobic contributions have been suggested and there are a few crystal structures that support these suggestions.^7,8^ However, it is hard to evaluate their importance in other sequential contexts and in the native aqueous environment in which glycoproteins function.

A few years ago, we began to use NMR, combined with long-range conformational restraints coming from paramagnetic entities, to search for preferred conformations of the simple N-glycan, GlcNAc_2_Man_5_, as it exists at the surface of a glycosylated protein.^9,10^ The N-terminal domain of the highly glycosylated Carcino Embryonic Antigen-related Cell Adhesion Molecule 1 (CEACM1-Ig1) was selected as a target. CEACAM1 is a molecule of significant biomedical interest because of its native role as an immune system regulator.^11^ However, its elevation in certain types of cancer also led to its early consideration as a biomarker for this disease and a potential target for therapy.^12,13^ It also acts as a receptor for pathogens, including *Helicobacter pylori* ^14^ and *Escherichia coli* strains causing gastrointestinal and urinary tract distress.^15^ The glycans certainly modulate some interactions of CEACAM1 with proteins of the human immune system and invading pathogens, but we focused on the interaction of glycans on CEACAM1 with its own surface, in the hope of providing experimental support for fundamental interactions that protect and stabilize the protein. With added support we hoped others might use knowledge of these fundamental interactions to engineer better versions of the numerous biologics now emerging from pharmaceutical research.^16,17^

The strategy that we adopted was to generate a pool of likely glycan conformations, using an accelerated version of molecular dynamics (MD), and then screen these conformations by comparing predicted and experimental NMR parameters. These parameters would include ones capable of positioning glycans at the protein surface, namely pseudocontact shifts (PCSs) and paramagnetic relaxation enhancements (PREs). We used mammalian cell culture to express CEACAM-Ig1 having a homogeneous set of GlcNAc_2_Man_5_ glycans with^13^C enriched at anomeric carbons as well as acetyl methyl groups, providing the necessary NMR active sites.^10^ A lanthanide ion binding loop was inserted in the protein sequence to provide PCSs and PREs. (see Figure 1A).^9^ Prediction of PCS data for various glycan conformations required evaluation of an anisotropic magnetic susceptibility tensor for the lanthanide ion used (Tb^3+^). Valine isotopically labeled with ^13^C in methyl groups was therefore added to the culture medium to provide data for the evaluation of this tensor; alanine methyls were additionally labeled from the C1-^13^C-labeled glucose used to label the glycans. Measurement of PCSs on the labeled protein sites along with an AlphaFold structure allowed determination of a susceptibility tensor and prediction of glycan PCSs. Nuclear Overhauser Effects (NOEs) measured experimentally and predicted based on the generated glycan conformers complimented the paramagnetic data.

**Figure 1:**
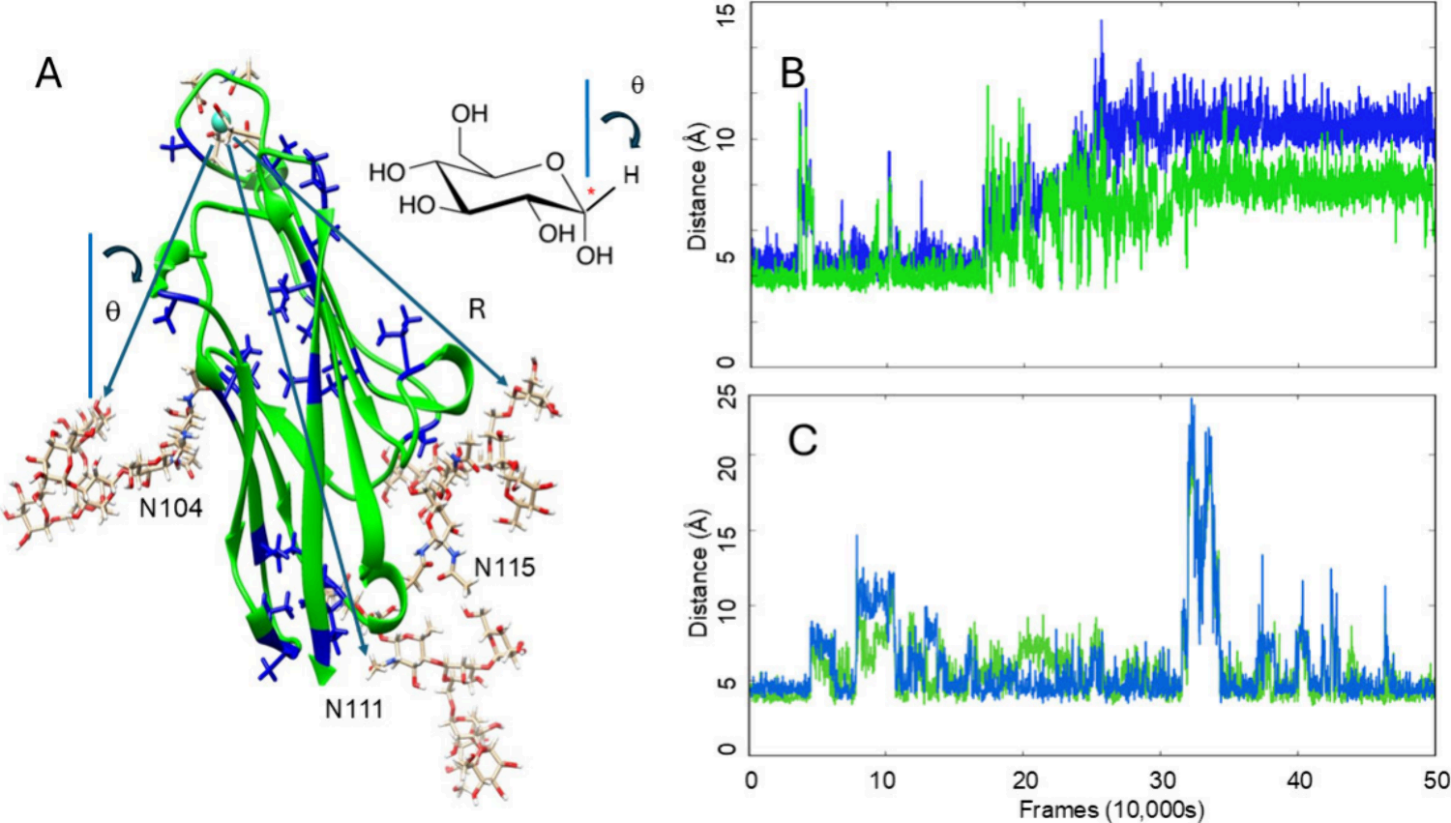
(A) CEACAM1-Ig1 construct. The lanthanide ion, bound to a 16-residue peptide loop inserted in the CEACAM sequence, is depicted in cyan. Blue arrows depict vectors of length, R, that connect the lanthanide ion to labeled sites on the attached glycans. The red * on the expanded glucose structure shows the site of ^13^C labeling on glucose. When C1­(^13^C)-labeled glucose is fed as a substrate, ^13^C ends up at the anomeric carbon of each glycan residue plus the acetyl methyls of the GlcNAc residues. PCSs provide both distance and angular constraints on the position of these glycan residues; RDCs provide constraints on directions of bonds at the labeled sites. (B) 1 μs conventional MD trajectory showing distances between the acetyl methyl of the second GlcNAc on N115 and surface atoms A77-Me (green) and P76-CB (blue). (C) The same distances are shown for a 1 μs Gaussian accelerated MD trajectory. Similar structures are sampled but at a rate 10 to 100 times more frequently.

Molecular Dynamics (MD) is a powerful computational method for biomolecular simulation but generating a trajectory of sufficient length to adequately sample glycan conformations, as they are attached to an N-glycosylation site, can be a challenge. We thus applied an enhanced sampling version of MD, namely Gaussian accelerated MD (GaMD), to generate trial structures of glycans along with a sampling of protein surface contacts.^18^ Figure 1B maps distances between the acetyl methyl of the first GlcNAc on the N104 glycan of CEACAM-Ig1 (UniProt P13688 numbering) and atoms of two protein surface residues, the A94 methyl carbon (green) and the P93 beta carbon (blue), during a 1 μs conventional MD simulation. The simulation begins with a set of close contacts (4–5 Å) with some short-lived excursions to 6–8 Å and a few beyond. At about 500 ns there is a large displacement to average distances of 8 and 11 Å for A94 and P93 respectively. There is no return to close contacts, and it is tempting to conclude that a more extended conformation is preferred.

While more advanced computational resources can extend sampling times by orders of magnitude,^19^ we chose GaMD to accelerate the simulations.^18^ This works by raising the minima of wells on an energy surface in a way that preserves the energy ordering of sampled structures while reducing the barriers to exchange between structures.^20^ Clustering glycan conformers and rescoring energies allowed estimation of cluster probabilities and selection of representative conformers to screen with PCS data. As a result, the glycan at the first N-glycosylation site (attached to N104) was suggested to adopt a conformer that maintained a close contact between the N-acetyl methyl of the first GlcNAc residue and a hydrophobic pocket containing leucine 54 and histidine 56. NOE contacts with chemical shifts consistent with leucine methyl protons and a histidine ring HD2 proton for one of the 6 acetyl methyls supported this suggestion. Hence, a role for hydrophobic interactions between protein and attached glycans in stabilizing protein structures and protecting surfaces from undesirable interactions with pathogenic entities was supported.

Despite the successful aspects of this initial investigation, the structural information returned by PCSs and PREs is limited to placing glycans in a general region of space near the protein surface, and surface contacts giving rise to short-range NOE data do not always involve groups that carry protons with uniquely assignable resonances. Hence, concerns remain both about the amount of NMR data collected and the adequacy of conformational sampling by the basic accelerated MD methodology. Fortunately, there have been advances in both NMR and MD methods that allow us to add to what we learned in our initial application.

For NMR, a third source of long-range structural data has existed for decades, namely residual dipolar couplings (RDCs).^21,22^ These arise from through-space dipolar interactions between pairs of NMR active nuclei and depend on angels θ and ϕ between an interaction vector (a ^13^C–^1^H bond vector of length, r, for example) and a frame of reference as illustrated in eq 1. A constraint on anomeric CH bond angles and acetyl CH_3_ bond angles would be of obvious utility. In our initial study, we did not pursue the measurement of RDCs because of limited resolution and sensitivity. But recently, an efficient pulse sequence exploiting J-modulation in a third dimension has been introduced,^23^ and additional modifications have now allowed us to make the necessary measurements (see Supporting Information).

RDCs normally average to zero under isotropic tumbling of molecules in solution, but these can be restored by partial orientation of the molecule of interest. In our system orientation comes from interactions between the static magnetic field and field-induced dipole moments. The moments are proportional to the anisotropic magnetic susceptibility (denoted simply as susceptibility from this point on) of the molecule as enhanced by the presence of a lanthanide ion (Δχ in eq 1). Note that the same susceptibility tensor elements are present in both the RDC equation and the previously used PCS equation (eq 2).^24,25^ Thus, no new variables are encountered upon adding RDC data. We have also doubled the amount of data available by using two different lanthanide ions, Tb^3+^ as was used in our initial study and Tm^3+^ which has a susceptibility tensor of smaller magnitude, opposite sign, and slightly different rhombicity.

1 $$\mathrm{RDC}:D_{CH}=-\frac{hB_{0}^{2}\gamma_{C}\gamma_{H}}{240\pi^{3}k_{B}Tr_{CH}^{3}}\left[\Delta \chi_{ax}\left(3\cos^{2}\theta -1\right)+\frac{3}{2}\Delta \chi_{rh}\sin^{2}\theta \cos2\phi \right]$$

2 $$\mathrm{PCS}:\delta^{pcs}=\frac{1}{12\pi R^{3}}\left[\Delta X_{ax}\left(3\cos^{2}\theta -1\right)+\frac{3}{2}\Delta X_{rh}\sin^{2}\theta \cos\left(2\phi \right)\right]$$

There have also been improvements in GaMD methodology. Two novel algorithms, Peptide-GaMD (Pep-GaMD) and Ligand-GaMD (LiGaMD), have been introduced.^26–28^ These were designed to improve the study of ligand/peptide dissociation and binding to proteins. They do this by applying a selective boost to either the essential potential energy of a peptide or the nonbonded potential energy of a ligand. They also include a “dual-boost” option that allows a complementary boost of the remaining potential energies. While our glycans are neither peptides nor ligands, the energies of glycan atoms can be selectively boosted in the same way that those of peptide or ligand atoms are boosted. Focusing on one glycan at a time minimizes the overall perturbation to the protein structure and allows a more selective ranking of individual glycan poses. We have chosen to collect Pep-GaMD data with energy boosts on each of the three CEACAM1-IgV glycans separately. We have also improved our clustering and rescoring procedures to calculate cluster probabilities and retrieve representative structures in a more streamlined fashion.

## Results

Trial structures for glycans were generated using Pep-GaMD separately for each of the three glycans on CEACAM1-Ig1 as described in the Methods section. One us trajectories were run in a box of TIP5P water molecules extending 10 Å beyond the limits of protein and glycan atoms. The choice of water model is an important one. TIP3P is more commonly used because it minimizes computation time. However, we noted the occurrence of numerous glycan–glycan interactions when using the TIP3P model that were not supported by experimental data. Tendencies for TIP3P to overemphasize interactions in intrinsically disordered proteins have been noted previously,^29,30^ and investigation of water models affecting glycosaminoglycan properties have suggested TIP5P to be among more suitable water models.^31^

Pep-GaMD simulation frames were saved every 2 ps and sampling every tenth frame gave 50,000 frames that were clustered based on the similarity of structures for the first two GlcNAc residues and the three protein residues at the attachment site. Clustering parameters were set to maximize the number of clusters having more than 500 members. This resulted in between 28 and 32 clusters for each site (N104, N111, and N115). The boost energies were removed as described in the literature,^20^ probabilities were assigned, and the low energy member of each cluster was selected as a representative structure. Probabilities are depicted in Figure 2.

**Figure 2:**
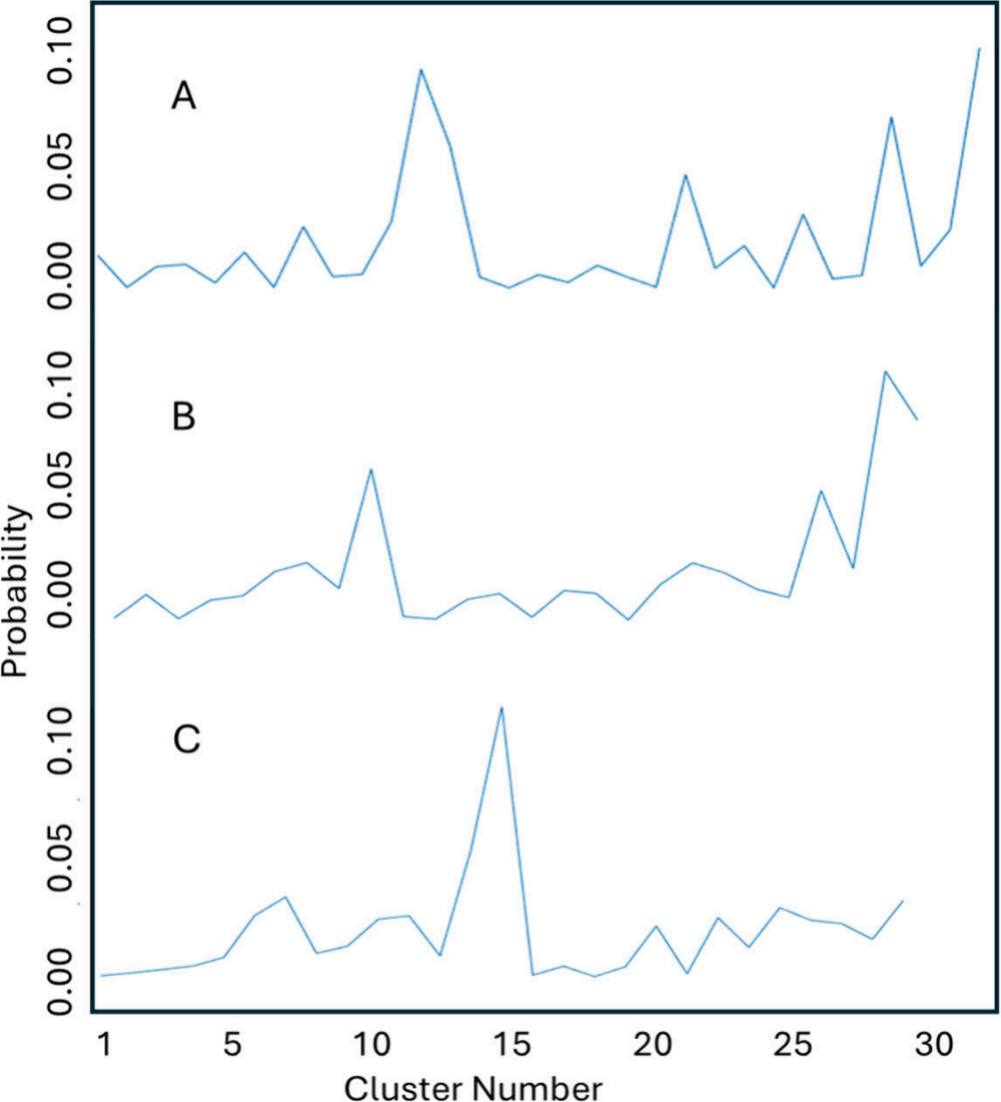
Relative probabilities for clusters of glycan structures. (A) N104 glycan. (B) N111 glycan. (C) N115 glycan.

In order to use PCS and RDC data in screening MD conformers for compatible structures of attached glycans, we need to first determine the Δχ values that occur in eqs 1 and 2. We accomplish this using a known protein structure along with PCSs and RDCs measured for a set of sites in the protein. As in our previous work we labeled valine methyls by including ^13^C enriched valine in the protein expression medium, and alanine methyls were labeled to ∼50% from the C1-^13^C glucose used to label the glycan residues. Measurement of PCSs simply consists of measuring the differences in chemical shift between a pair of ^13^C-detected 2D HSQC spectra, a reference spectrum for a protein complexed with a diamagnetic Lu^3+^ ion and a spectrum for a protein complexed with a paramagnetic ion (Tb^3+^ or Tm^3+^ in our case). In this work, measurements were facilitated by the application of a MATLAB app that displayed connections of crosspeaks and recorded data in EXCEL spreadsheets (Supporting Information).

Measurement of RDCs was performed by analyzing the modulation of crosspeak intensities in a ^13^C-detected pseudo-3D J-modulated constant time HSQC spectrum as described further in the Methods section and Supporting Information. Initial measurements included 22 PCSs and 15 RDCs for protein sites in the Tm^3+^ sample and 17 PCSs and 13 RDCs for protein sites in the Tb^3+^ sample. However, GaMD trajectories showed significant internal motion for valines 45, 73 and 80, as well as alanine 34. Suspecting that experimental RDC values assigned to these sites would be affected by motional averaging, we iteratively excluded data from these sites, along with two clear outliers, during the resonance assignment process. This resulted in sets of 20 PCSs and 11 RDCs for Tm^3+^ and 14 PCSs and 7 RDCs for Tb^3+^. Analysis proceeded separately for the Tb^3+^ and Tm^3+^ data.

A modified version of the ASSIGN_SLP program^32^ was used to simultaneously make assignments of crosspeaks and determine parameters from which Δχ values can be calculated. The program was initially designed to derive resonance assignments from a combination of RDC, NOE, and chemical shift data, but it has been modified to also consider PCS data, as well as export variables used for susceptibility tensor determination. The program calculates values corresponding to these observables from a known structure of the protein and compares these values to observables to make assignments. While there are crystal structures of the CEACAM1-Ig1 domain, the addition of the lanthanide-binding loop dictated using a computationally derived structure (Figure 1A); it was generated in our previous work using AlphaFold.^29^

The output of magnetic susceptibility information was in the form of five independent Cartesian variables from which the five independent elements of a traceless and symmetric susceptibility tensor were calculated. The tensor properties are easily visualized by plotting PCS isosurfaces at selected constant PCS values. This is illustrated in Figure 3 for an isosurface plotted at −0.13 ppm for Tb^3+^ + 0.08 ppm for Tm^3+^. Positive and negative PCS surfaces are colored blue and red, respectively. The surfaces reach a maximum distance from the paramagnetic ion at θ = 0 and r = 40 Å of their respective spherical coordinate principal alignment frames. Concentric PCS surfaces could be plotted at values that vary as 1/r^3^ (at 20 Å PCS values would be −1.04 and 0.64, respectively).

**Figure 3:**
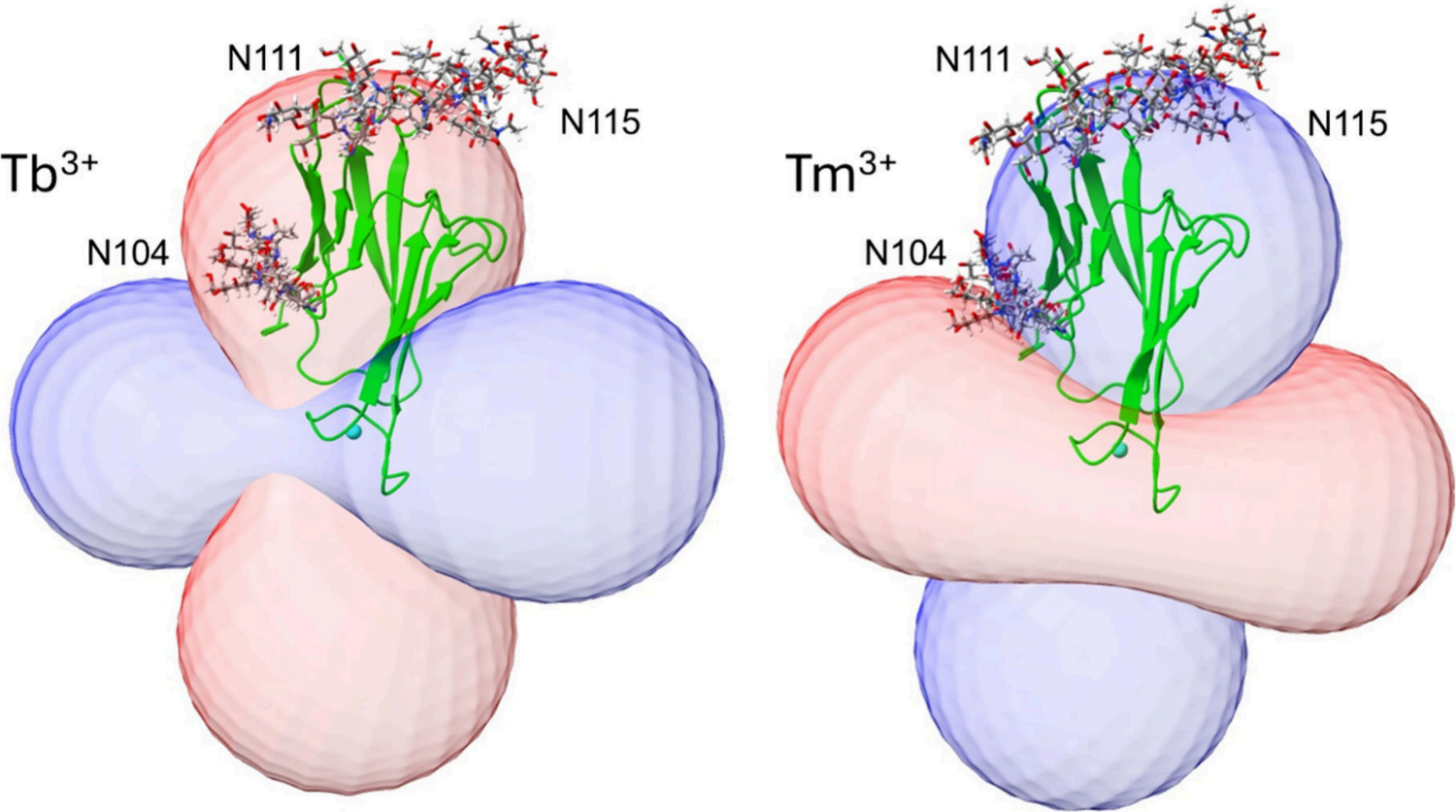
PCS isosurfaces for Tb^3+^ and Tm^3+^ complexes. Ribbon diagrams for CEACAM1-Ig1 structures have been put in the principal frames of the respective susceptibility tensors, and the combined isosurfaces and structures have been oriented to produce similar views of the ribbon diagrams. The four most probable structures for the first two GlcNAcs at each glycosylation site are also displayed.

For the four most probable clusters at each site, representative structures of the first two GlcNAcs plus the linking asparagine were superimposed on the CEACAM1-Ig1 structure shown in Figure 1 by matching the protein backbone atoms. These superpositions are also displayed in Figure 3.

There are a couple of things to note. First, only one of the three sites, N104 (lower left), has glycans primarily inside the depicted surfaces. This is especially apparent in the Tb^3+^ complex. The steep 1/r^3^ dependence of PCSs on distance from the lanthanide ion (cyan sphere) suggests that we can assign crosspeaks to the N104 glycan based on the large magnitude of PCSs. Second, the superimposed structures cluster more tightly for the N104 site than for either the N111 or N115 sites. This suggests that restraining glycan-protein interactions are more prevalent for the N104 site. A quantitative comparison of experimental PCS and RDC values to values predicted from each of the representative structures should shed light on the nature of these interactions.

For screening of the of the glycan conformations both, PCSs and RDCs were measured. As with the protein data, these relied on isotopic labeling with ^13^C, in this case anomeric carbons of all glycan residues (C1–H1 pairs) and acetyl methyls of the GlcNAc residues. We will focus on the GlcNAc residues because of the extensive variation in conformations of the more terminal mannose residues. Crosspeaks for the C1–H1 pairs can be assigned to either the first or second GlcNAc based on unique chemical shifts.^33^ Shifts differ both because of the residue type and the differences in linkage. The first GlcNAc at each site is linked to the amide nitrogen of an asparagine side chain (3 data points), while the second GlcNAc at each site is linked to the O4 of the first GlcNAc (3 data points). Acetyl methyls are present only on the GlcNAc residues, but the corresponding crosspeaks cannot be assigned to a specific GlcNAc based on chemical shifts alone (6 data points). Specific assignments will be made later based on agreement between predicted and experimental shifts.

Glycan PCSs were measured as described above for protein data and in our previous publication. Glycan RDCs were measured using the same J-modulated spectra that were used for the protein RDCs (see Methods and Supporting Information sections). An example of RDC data coming from one of the anomeric C1–H 1s pairs in an O4-connected GlcNAc (2nd GlcNAc) is presented in Figure 4. Observed couplings correspond to the sum of dipolar (D) and scalar (J) couplings for paramagnetic complexes and just the scalar coupling for diamagnetic complexes. Hence, the RDC (D) is extracted by taking the difference in coupling measured in the presence of a paramagnetic ion (Tb^3+^ in this case, Figure 4A) and in the presence of a diamagnetic ion (Lu^3+^), resulting in an RDC of 8.1 Hz. The estimated errors come from the least-squares fit to the modulation. Since all scalar couplings for a particular residue and linkage type are expected to be the same, all Lu^3+^ data for a given type are averaged to reduce the diamagnetic error; total estimated error for this case ∼1.6 Hz.

**Figure 4:**
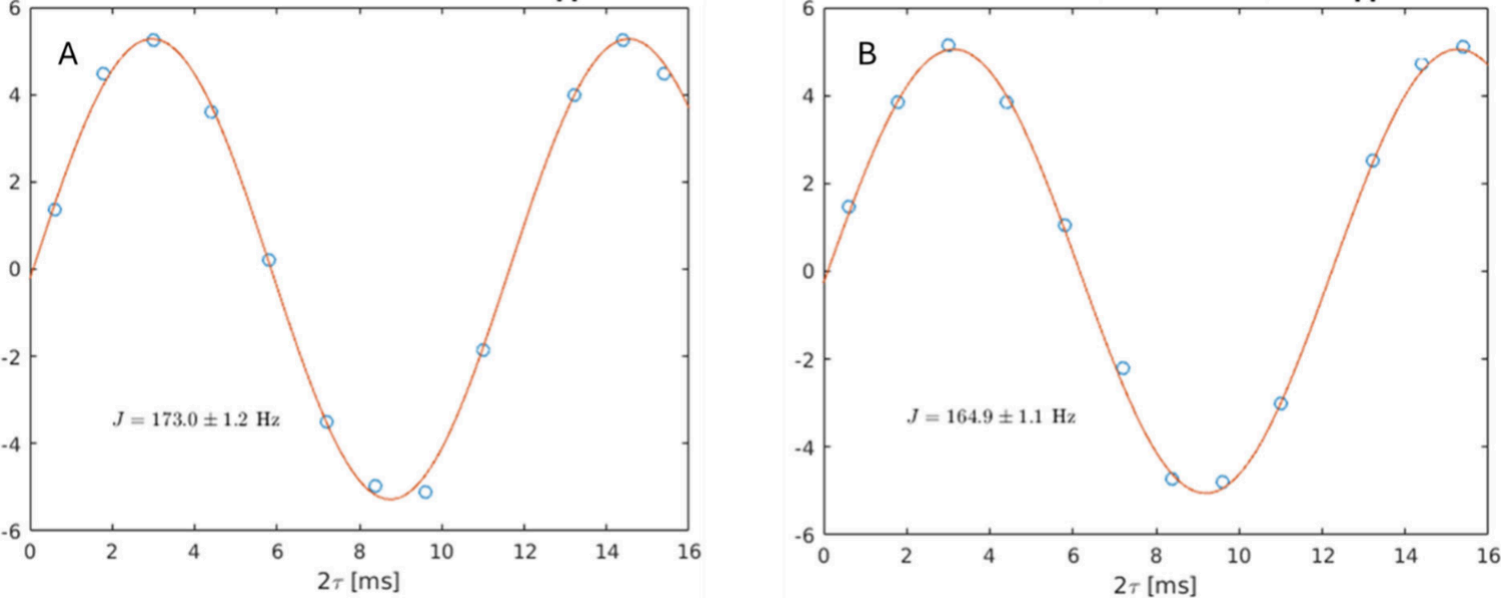
RDC measurement for the C1–H1 pair of a 2nd GlcNAc in Tb^3+^ (A) and Lu^3+^ (B) containing samples. The measured RDC (D) is the difference in couplings (J+D and J) measured in the two plots (+8.1 Hz).

As with the protein data, not all glycan RDC and PCS data could be measured because of paramagnetic line broadening or excessive peak overlap. In total 11 glycan PCSs and 8 glycan RDCs were used for the Tm^3+^ complex and 11 glycan PCSs and 7 glycan RDCs were used for the Tb^3+^ complex. Resonance assignment possibilities are limited by ^13^C isotope labeling only of anomeric and acetyl methyl sites and by our choice to focus on the first two glycans (GlcNAcs) on each of the three glycosylation sites (potentially 12 PCSs and 12 RDCs). As RDCs are measured from the same shifted crosspeak as the PCSs, these must be assigned in pairs to a particular site. In principle, assignments could be made simply by comparing experimental PCS/RDC data pairs with pairwise values predicted using eqs 1 and 2. However, we expect a certain level of conformational averaging which makes use of RDC values particularly challenging. However, if averaging is limited, as suggested for the N104 site (see Figure 3), it should be possible to pick a best match to predictions using a single structural representative, or possibly an average over predictions from two or three of the more probable conformers (see Figure 2).

Tables 1 and 2 present a comparison of experimental RDC-PCS pairs to single structure predictions using Tm^3+^ and Tb^3+^ susceptibility tensors, respectively. Pairs are listed with the PCS first and the RDC directly below. For predictions we consider only the representative structures from the four highest probability clusters at each site. The predictions are ordered by cluster probabilities within each site (highest probability first) and identified by the frame number of respective trajectories. The tables are color coded with a different color for each site; blue for N104, peach for N111 and green for N115. The experimental data are entered adjacent to the best matched set of predicted values, and matches are highlighted by darkening the colors. There are three columns for experimental data; first C1 denotes the anomeric CH of the first GlcNAc at each site (distinguished by chemical shift, 3 entries), second C1 denotes the anomeric CH of the second GlcNAc at each site (distinguished by chemical shift,3 entries), Ac denotes the acetyl methyls, which cannot be assigned to a specific GlcNAc (6 entries). One first C1 must be assigned to each glycan site, one second C1 must be assigned to each site, and two Acs must be assigned to each site.

**Table 1:**
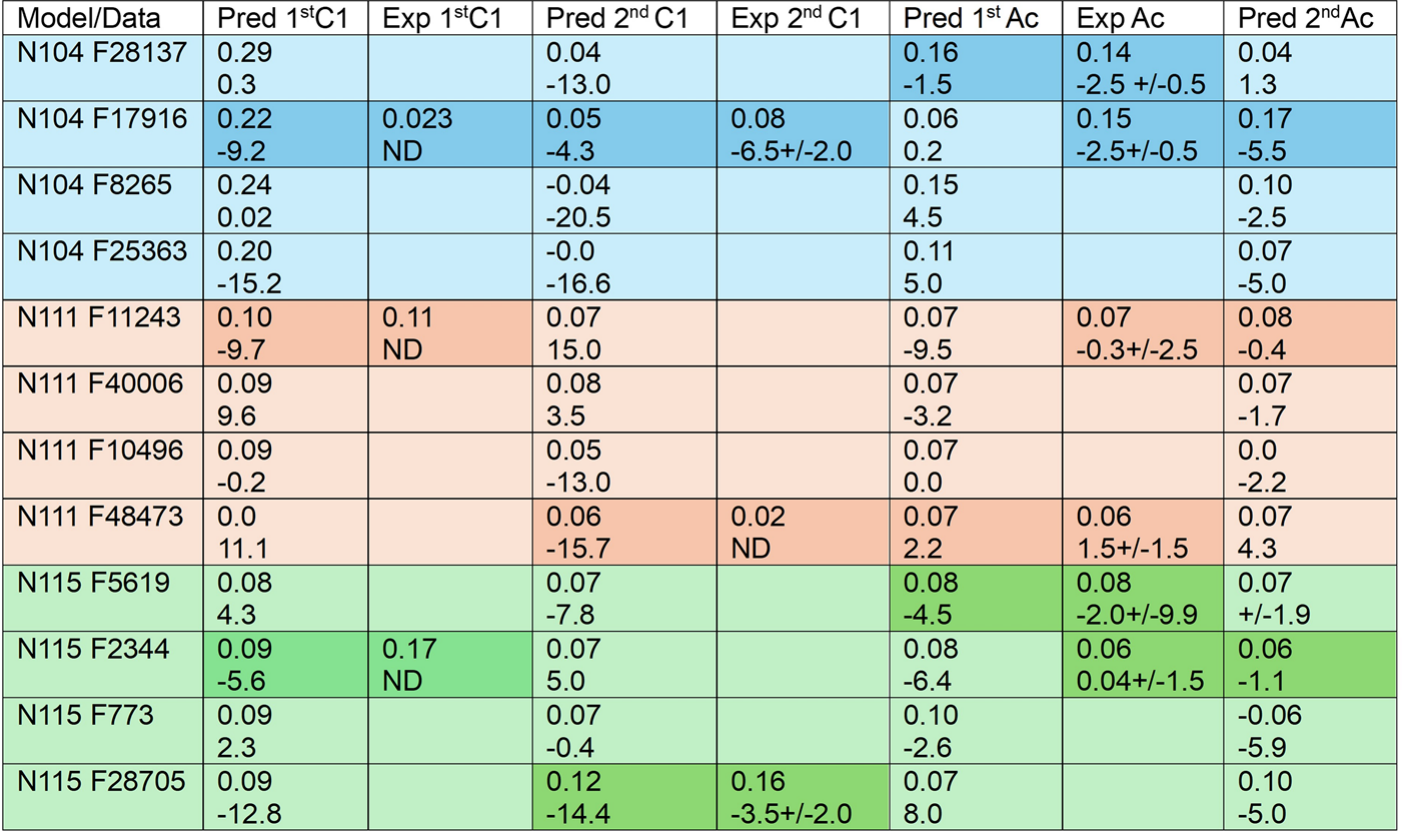
Analysis of Tm^3+^ Glycan Data^a^

a Table 1 compares experimental (Exp) to predicted (Pred) PCS and RDCs Tm^3^ data for glycans at the 3 glycosylation sites of CEACAM1-Ig1. PCSs (ppm) and RDCs (Hz) are presented in pairs as they originate from the same HSQC crosspeak. Estimated errors for experimental PCS are 15% with a minimum of 0.03 ppm (∼line width). RDC errors are from least-squares fits of data. ND denotes not determined.

**Table 2:**
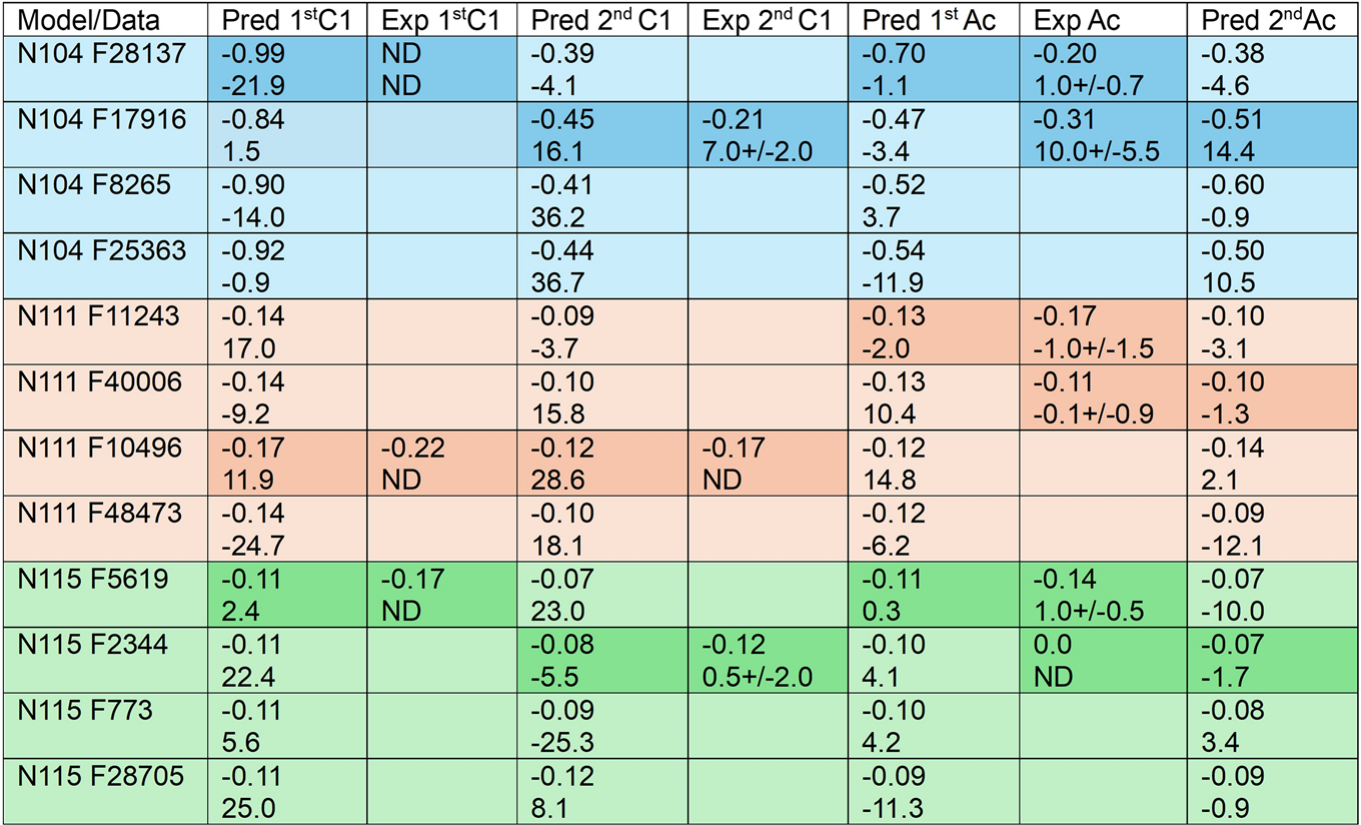
Analysis of Tb Glycan Data^a^

a Table 2 compares experimental (Exp) to predicted (Pred) Tb^3^ PCSs and RDCs for glycans at the 3 glycosylation sites of CEACAM1-Ig1. PCSs (ppm) and RDCs (Hz) are presented in pairs as they originate from the same HSQC crosspeak. Estimated errors for experimental PCS are 15% with a minimum of 0.03 ppm (∼line width). RDC errors are from least-squares fits of data. ND denotes not determined.

For the N104 site in the Tm^3+^ table (Table 1, blue rows), the structure from the GaMD frame 17916 (cluster 12 representative) provides an acceptable match (within 2 x experimental error) for nearly all PCSs and RDC data. The exceptions are the first Acetyl RDC, where the structure from frame 28137 provides a better match, and the second acetyl, where the experimental RDC deviates from predictions by more than 2× estimated error. Clearly there is some averaging over structures. In some cases, averaging over predictions from the two most probable structures can improve agreement. For example, a 50:50 average over predictions from frame 28137 and frame 17619 for the second GlcNAc acetyl RDC results in a value of −2.1 Hz, well within error estimates for the experimental value of −2.5 Hz. Averaging likewise improves the agreement for the second GlcNAc C1 RDC. Most PCSs for N104 stay within acceptable error bounds under this type of averaging. There may well be some additional averaging over structures not represented by the four most probable clusters that would further improve fit to data. In principle, it would be possible to use a larger ensemble of structures along with probabilities assigned to the clusters they represent. However, the energies on which the probabilities are based are not sufficiently accurate to pursue this course of analysis, and there is not sufficient data to include multiple probabilities as additional variables. Nevertheless, the degree to which sampling just two structures fits the data suggests considerable restraint of conformational sampling due to glycan–protein interactions. The fact that some of the experimental RDCs deviate significantly from the zero value expected under isotropic averaging, reinforces this suggestion. Based on the magnitude of anisotropic susceptibility tensors determined from protein data, C1–H1 RDCs would range from approximately +25 to −25 Hz and acetyl methyl RDCs about 1/3 that. This range corresponds to a change in vector direction of just 90 deg, so RDC data provide a very stringent test of structural compatibility and motional averaging.

For the N111 (peach) and N115 (green) sites, predicted PCSs are more uniform and near 0.08 ppm. This is consistent with the distribution shown in Figure 3B where all N111 and N115 glycans are near the blue surface drawn at ∼0.08 ppm. Predictions of PCSs for the glycans at site N104 are more variable due to their position near the transition from positive (blue) to negative (red) PCS surfaces. For the N111 and N115 there is one moderately large and positive predicted PCS for a second GlcNAc C1 (0.12 ppm) that matches the largest experimental value of 0.16 within acceptable experimental error and allows a definitive assignment, but similarity of other PCSs prevents other definitive assignments based on PCS values alone. The predicted RDCs show a wide range of values (both positive and negative) for structures representing the clusters for these sites. The experimental RDCs are generally smaller, suggesting the need for averaging over several predicted values to fit the data. This is consistent with glycans attached to the N111 and N115 sites experiencing less restriction due to interaction with the protein surface.

Table 2 presents data collected on a sample having Tb^3+^ in the lanthanide binding site. The data are again compared to predicted values using coordinates from the same representative structures. Tb^3+^ is expected to have susceptibility components that differ in sign and are ∼60% larger than those for Tm^3+^. Thus, both RDC and PCS are expected to be correspondingly large, and likely include a sign change.^24^ Unfortunately, contributions to paramagnetic relaxation enhancement are somewhat larger for Tb^3+^, and chemical exchange broadening of peaks due to PCS variation will be about 2.8 times larger. These effects result in some additional loss of data, both for glycan sites and protein methyls used to extract susceptibility tensors. The determination of the Tb^3+^ susceptibility tensor used just 7 RDC values and 14 PCS values from protein sites compared to 11 RDCs and 20 PCSs for the Tm^3+^ susceptibility tensor. Nevertheless, general trends are expected to be complementary.

For the N104 site, deviations of experimental from predicted PCS values are large, but the best fits are again to the frame 17916 and 28137 structures. PCSs predicted for the N104 site are in all cases, larger than those predicted for the other sites. This is consistent with Figure 3A in which the first two residues of the N104 glycan models are all well inside the −0.13 ppm PCS surface for the Tb^3+^ data. Hence, assignment of crosspeaks having the largest magnitude of PCS within each chemical class to the N104 site seems appropriate. Acetyl crosspeaks with PCSs of −0.31 and −0.20 are assigned to the first and second acetyl of the N104 glycan. Likewise, the largest PCS for the second GlcNAc C1–H1 group (−0.22 ppm) is assigned to the N104 site. A suitably large PCS was not observed for first GlcNAc C1–H1 group, possibly due to exchange broadening of the peak. Averaging predictions for RDCs for the second C1–H1 and second acetyl over predictions from the frame 28137 and frame 17619 structures would improve fits to the RDCs

For the N111 and N115 sites, predicted PCS values are too similar to make confident assignments. However, best matches upon including RDC data are presented. Using the combined data, assignments are spread over representative structures from 3 and 2 of the highest probability clusters. There are several cases where averaging over the structures selected improves the fit to experimental RDC data. For example, averaging over structures from frames 40006 and 10496 for the second acetyl at site N111, and averaging over structures from frames 5619 and 2344 for the first acetyl at site N115 improves the fit. It is interesting that none of the RDCs assigned to N111 or N115 sites reach the magnitudes of RDCs assigned to N104 sites. This is consistent with more extensive motional averaging at these sites, as uniform sampling of bond directions reduces RDCs to zero. If there are strong conformational preferences imposed by protein surface contacts, they are likely to occur for the glycan attached to the N104 site.

In principle, there are additional data on contacts between glycans and the protein surface coming from Nuclear Overhauser Effects (NOEs). Interpretation normally requires specific assignments of both receiving and donating protons. Here we can begin with the associations in Tables 1 and 2 for the N104 site, along with PCS connections to the Lu^3+^ spectra, to make assignments of receiving crosspeaks. We then suggest donor proton types based on chemical shifts. For example, a crosspeak at 25.4 ppm for ^13^C and 1.93 ppm for ^1^H in the Lu^3+^ sample would be assigned to the second GlcNAc acetyl methyl at the N104 site and a crosspeak at 25.0 ppm for ^13^C and 1.93 ppm for ^1^H would be assigned to the first GlcNAc acetyl methyl at this site. The crosspeak at 25.4 and 1.93 ppm shows a strong NOE to a proton with a 0.70 ppm shift and a weaker NOE to a proton with a 0.80 ppm shift. These shifts are typical of leucine or valine methyls. It also shows a strong NOE to a proton with a shift of 6.8 ppm. This is typical of an aromatic or histidine HD2 proton. The crosspeak at 25.0 and 1.93 ppm also shows a strong NOE to a proton with a 0.70 ppm shift, one typical of leucine or valine methyl and a weaker NOE to a proton with a shift of 1.2 ppm, typical of a methylene in an aliphatic amino acid or alanine methyl. It also shows a weak NOE to a proton with a shift of 6.6 ppm, typical of an aromatic or a histidine HD2 proton.

## Discussion

The clustering of high probability structures, and the reasonable agreement with data on averaging just a few structures, suggests taking a closer look at structures predicted for the glycan attached to N104. Figure 5 depicts the first two GlcNAcs and the nearby protein surface residues from frame 28137 (A), which is very similar to the structure identified in our prior publication, and frame 17916 (B), which matches more of the current data and differs somewhat from structures considered in our prior publication. The inclusion of this second structure likely results from the broader search executed here using Pep-GaMD, and its selection results in part from its agreement with new RDC data. PCSs predicted for these structures are uniformly large and negative for Tb^3+^. However, the predictions are more varied for Tm^3+^ and agreement with the new experimental data favor the structure from frame 17916. As depictions of both structures suggest specific, but different, interactions with surface residues, a closer examination of the extent to which experimental data constrain those structures is useful.

**Figure 5:**
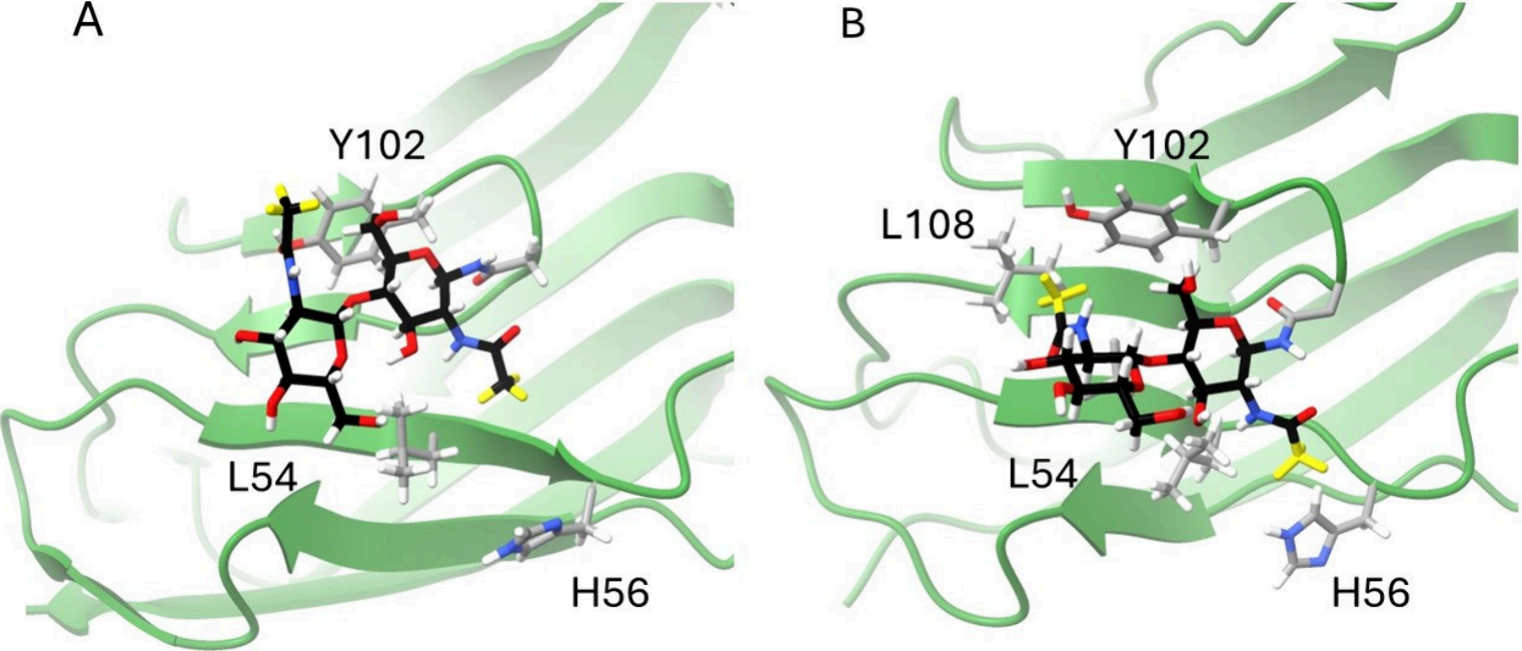
Glycan conformations from representative structures for cluster 31, frame 28137 (A) and cluster 12, frame 17619 (B). The first two GlcNAcs are depicted along with protein residues showing potential contact with glycans. Carbons in the glycans are colored black to provide contrast. Labeled sites (acetyl methyls and C1–H 1s) are colored yellow.

PCSs primarily offer a constraint to a general region close to the protein surface. However, RDCs are very sensitive to bond directions and can illustrate better sensitivity to glycan structure. We can point to some examples of this sensitivity by comparing bond directions in the structures of Figure 5 to predicted data in Tables 1 and 2. This is most appropriate if one of the couplings is fairly large, indicating a bond angle near the limits of its (3cos^2^(θ) – 1) dependence. For example, the two C1–H1 bond vectors of the GlcNAcs in Figure 5B (frame 17619) appear to point in nearly opposite directions (180 deg), while the two acetyl methyls appear to point at right angles to one another. If one of the RDCs is fairly large, we would expect the two C1–H1 RDCs to be of the same sign and the two acetyl methyl RDCs to be of different signs. In the Tb^3+^ predictions (Table 2) the second acetyl RDC is large and positive (14.4 Hz) and the RDC for the first acetyl is of the opposite sign (−3.4 Hz) as expected. The second C1–H1 has a large RDC (+16.1 Hz) and the 1^St^ C1–H1 has a positive RDC (+1.5 Hz) as expected. In the Tm^3+^ predictions (Table 1) the first C1–H1 has a fairly large negative RDC (−9.2 Hz). The second C1–H1 has an RDC of the same sign (−4.3 Hz), as expected. In Figure 4A (frame 28137) the first and second C1–H1 bonds also appear to point in opposite directions. In the Tb^3+^ predictions (Table 2), the RDC for the first C1–H1 is large and negative (−21.9 Hz) and the RDC for the second C1–H1 is of the same sign (−4.1), as expected. Experimentally RDCs are affected by averaging over structures, so the correlations are not always as clear, but they do qualitatively support the predicted correlations. For example, in Tb^3+^ data for frame 17916 (Table 2) a large positive RDC (7.0±2.0 Hz) is assigned to the second C1–H1, and in Tm^3+^ data for frame 17619 (Table 1) a large negative RDC (−6.5±2.0 Hz) is assigned to the second C1–H1.

The close contact with protein surface residues depicted in Figure 5 is significant. Based on Tb^3+^ PCS data in our previous publication,^9^ the contact between the first acetyl group of the N104 glycan and a pocket containing leucine 54 and histidine 56 was suggested to be a stabilizing hydrophobic interaction. Its existence was supported by NOE contacts between an acetyl group and protons with chemical shifts typical of a histidine HD2 proton and leucine methyl. The additional Tm^3+^ PCS data and RDC data for both Tb^3+^ and Tm^3+^ complexes now confirm this interaction. Both structures in Figure 5 show this contact.

The additional contact between the acetyl methyl on the second GlcNAc and a pocket containing tyrosine 102 and leucine 108 depicted in Figure 5B is new, and it too suggests the importance of a hydrophobic interaction. Some aspects of this interaction may be shared more widely with other glycoproteins. Motifs in which an aromatic residue is at a −2 position relative to an N-glycosylation site have been noted before and are termed an “enhanced aromatic sequon”.^7^ Tyrosine 102 is in the −2 position relative to asparagine 104. In addition to hydrophobic interactions, C–H−π bonding to the aromatic surface has been suggested.^8^ Tyrosine 102 also proves to be sufficiently close to the H1 proton of the more hydrophobic surface of the first GlcNAc in Figure 5A to make this type of contribution. While C–H−π bonding may not be the dominant force driving interactions in the present case, the occurrence of an enhanced aromatic sequon in CEACAM1-Ig1 and other glycoproteins suggests a functional importance.

The role of additional hydrophobic contacts made by acetyl methyls is also of interest. Despite the diversity of fully elaborated N-glycans, all N-glycans start with a pair of N-acetylated glucosamine residues. Also, complex O-glycans typically start with an acetylated sugar, N-acetyl galactosamine, and addition of a single N-acetylated glucosamine to targeted serine or threonine residues is now recognized as a master regulator that competes with phosphorylation.^34^ It is likely that acetyl methyl hydrophobic contacts with protein surface residues play a role in the function of most types of protein glycosylation.

The N111 and N115 sites do not have the same prevalence of hydrophobic patches near the attachment site that the N104 site (A) does. Figure 6 depicts these surfaces colored by lipophilic tendencies (gold most and blue least). The first GlcNAc in the conformation of the most probable cluster is shown at each site. The N104 site has a significant hydrophobic surface, especially to the left of the attachment site. This type of surface is less prevalent in the N111 (B) and N115 (C) sites. Looking at the trajectories, there are occasional surface contacts for the N111 and N115 glycans, but they are far less numerous than the contacts made at the N104 site. As pointed out earlier, the representative structures for the N111 and N115 sites also are more dispersed than those for the N104 site. Hence, there appears to be very specific sites where hydrophobic interactions occur.

**Figure 6:**
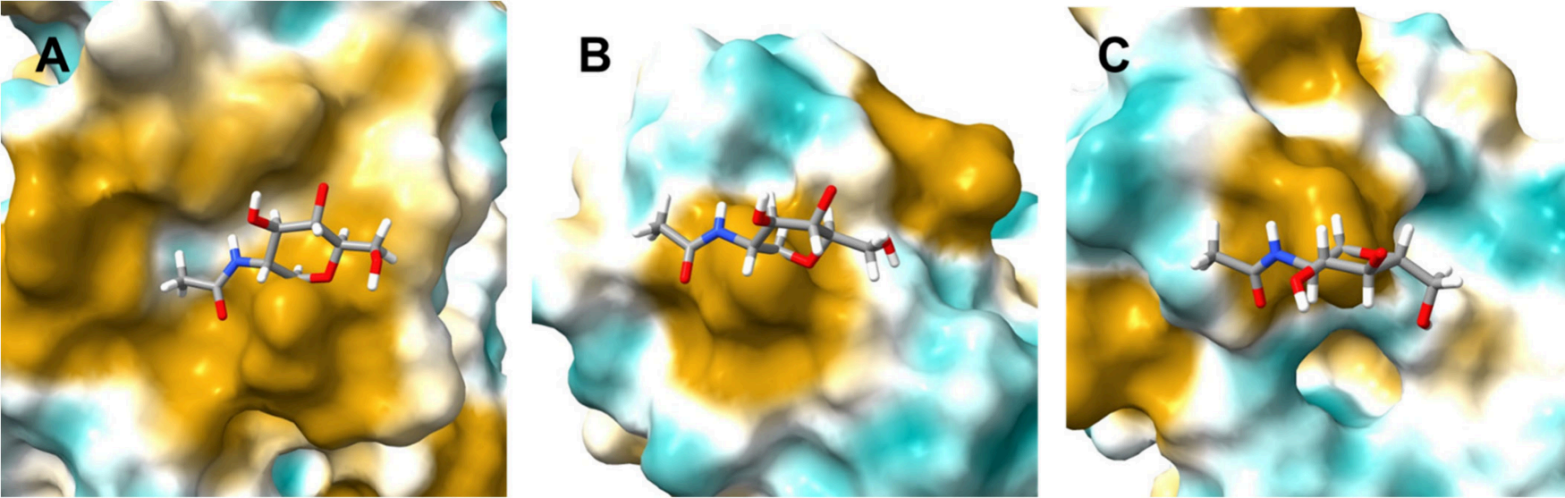
Surface plot showing the first GlcNAc at each of the glycosylation sites (N104 (A), N111 (B), N115­(C)) along with the underlying protein surface. Coloring is gold: most lipophilic or most hydrophobic, blue: least lipophilic or most hydrophilic.

The importance of these interactions can only be assessed by studies on additional systems that include functional changes in response to altering the composition of the interacting surfaces. However, additional systems having the types of interactions described here, as well as other types of surface interaction, need to be identified. The long-range NMR data from RDCs and PCSs, combined with NOEs showing contacts characteristic of protein residues has proven useful in the CEACAM-Ig1 case and can easily be applied in the search for other systems having surface contacts. Identification of new systems may also come from computational studies such as those using GaMD. Identifying conformers having stabilizing surface contacts does depend on the quality of the force fields describing interactions among proteins, glycans, and the water in which these systems function. Force field development is an ongoing process, but it is noteworthy that the glycan conformers showing the best fits to NMR data in the CEACAM-Ig1 case are from the GaMD clusters having the highest predicted populations. This offers much promise for the future.

## Methods

### Sample Preparation

Sample preparation follows procedures defined more fully in our previous publications.^9,10^ But briefly, a construct based on the sequence for the first IgV-like domain of human CEACAM1, residues 34–141 of the UniProt sequence P13688-1 was inserted into a pGEn2 expression vector. A 16-residue lanthanide binding loop, stabilized by the formation of a disulfide bond between terminal cysteine residues, replaced four residues in a beta turn connecting residues 83 and 88 in the initial sequence. Expression occurred in HEK293S (GnTI^–^) cells (ATCC) cultured in 1 L of a custom version of FreeStyle 293 media (Gibco, ThermoFisher Scientific). This medium, which lacked both glucose and amino acids, was supplemented with 5 g of ^13^C1-glucose, and 150 mg of ^13^C-dimethyl-valine (Cambridge Isotope Laboratories, Tewksbury, MA) along with other unlabeled amino acids normally present. Metal affinity chromatography was used to retrieve expressed product from the medium. The product was treated with TEV protease to remove a His tag and a GFP fusion sequence. Further purification by metal affinity chromatography and Superdex-75 gel filtration led to a final yield of ∼ 10 mg. The protein sequence and glycosylation level (90%) were verified by mass spectrometry. The particularly high level of glycosylation appears to have been facilitated by the addition of the disulfide bond in the lanthanide binding loop.

For NMR spectroscopy, the protein was exchanged into a pH 7.4 buffer composed of 25 mM Tris, 100 mM NaCl. Three samples in 5 mm Shigemi NMR tubes were prepared at 300 μL volume each and 250 μM protein concentration, containing 0.02% NaN_3_, 10 μM DSS, 10% D_2_O, and a 1:1 mol equiv of LuCl_3_, TmCl_3_, or TbCl_3_.

### NMR Data Collection and Analysis

NMR data acquisition was performed at 25 C using a 900 MHz Bruker AVANCE NEO spectrometer equipped with direct-observe TXO cryogenic probe, optimized for ^13^C/^15^N detection. For PCS measurement, 2D ^13^C-detected HSQC spectra were recorded for Lu^3+^, Tb^3+^, and Tm^3+^ complexes. The pulse sequence from Bruker Topspin library was modified to include semiconstant time (SCT) ^1^H frequency labeling for improved sensitivity. It also utilized an adiabatic ^13^C inversion pulse (20% smoothed CHIRP pulse with 80 kHz sweep, 500 μs pulse width) to allow simultaneous detection of glycan C1 and methyl signals in a single spectrum. ^13^C-detected HSQC was employed to take advantage of higher signal dispersion in the ^13^C dimension and reduced paramagnetic line broadening impact on ^13^C, in addition to avoiding H_2_O line interference with anomeric signals. At high fields and in the presence of paramagnetic ions the loss in sensitivity compared to normal ^1^H detection is modest (a factor of ∼2). 2048 and 160 complex time-domain points were collected at spectral widths of 118.8 and 7.6 ppm for ^13^C and ^1^H dimensions, respectively. A one s recycle delay was used, along with 64 steady-state scans, and 16 (Lu^3+^ and Tm^3+^ samples) or 32 (Tb^3+^ sample) scans per increment, resulting in acquisition times of, respectively, 1.6 or 3.2 h per spectrum.

PCS data were extracted from the resulting spectra by connecting crosspeaks in Lu^3+^–Tm^3+^ or Lu^3+^–Tb^3+^ pairs of spectra with diagonal lines in a ppm by ppm plot and averaging the differences in the chemical shifts for the two dimensions. This process was facilitated with a MATLAB script included in Supporting Information. RDC sets were collected on both Tm^3+^ and Tb^3+^ complexes, using a modified version of a J-modulation pulse sequence.^23^ As in prior works, ^13^C–^1^H J-modulation occurred during a constant time element that suppressed undesired ^1^H–^1^H J-modulation.^35^ For the paramagnetic complexes modulation is the sum of scalar (J) and residual dipolar (D) contributions. A reference set modulated only by scalar coupling was collected on a Lu^3+^ complex, and the difference in modulation frequencies was used to extract RDCs. Estimated error comes from the least-square fit to the modulation. An example of data and fit to the data is presented in Figure 4. The pulse sequence and acquisition parameters are described more fully in Supporting Information.

Assignment of crosspeaks coming from ^13^C labeled methyls of valine and alanine, as well as the export of variables used to calculate susceptibility tensor elements was done using a version of our sparse-label assignment software, ASSIGN_SLP, that uses a combined set of RDCs and PCSs as well as chemical shifts, paramagnetic relaxation enhancements (PREs) and NOEs.^32^ This version is available for download from our Web site (https://tesla.ccrc.uga.edu/software) and is available on NMRbox.^36^

### Gaussian Accelerated MD

The glycan structures to be screened for consistency with NMR data come from 1 μs MD trajectories performed using Pep-GaMD in AMBER22. The starting point for MD calculations was a minimum energy structure for the glycosylated protein selected from MD trajectories used in our previous work.^9^ The system was solvated in TIP5P water in a rectangular box with a minimum distance between the glycans and the edge of the box of 10 Å. The ff14SB force field was used for amino acids and the GLYCAM_06j-1 force field was used for glycans. After minimization, the system was heated to 300 K and allowed to equilibrate. The potential energy of one of the glycans and the remaining system potential energy were boosted in a dual-boost Pep-GaMD simulation, which began with a 4 ns conventional MD run and then 16 ns Pep-GaMD equilibration. In each of three 1000 ns Pep-GaMD production runs, simulation frames and energies were saved every 2 ps, resulting in 500,000 frames. The standard deviation of the boosts was set to 8.0 kcal/mol for all systems. It is important to note that probabilities assigned to these structures are ultimately dependent on the accuracy of force fields for glycans, protein and the particular water model used.

Sets of structures composed of every tenth frame in the original trajectories were clustered based on the root-mean-square deviation (rmsd) of the position of central ring atoms (C1, C2, C4, C5) of the first two GlcNAc residues and Cα carbons of the three residues at the glycan attachment sites. The Ward-Linkage Algorithm (ward) of the MATLAB 2024b linkage command was used to prepare the initial files containing pairwise rmsds (d file) and a Z-ordered description of this file. Subsequently, the minimum cluster size was set to 500 and an inconsistency cutoff was adjusted to produce the largest number of clusters. Probabilities for each cluster were calculated using cumulant analysis of exponential energies followed by removal of boost energies and calculation of a Boltzmann probability.^20^ The minimum energy structure for each cluster was then selected as a representative frame. This procedure was used in our previous study and the MATLAB scripts and MATLAB apps implementing the procedure were provided in supporting material for that study.^9^ Figure 2 shows screenshots of probability profiles for trajectories in which energies for each of the three glycans were selectively boosted. In our subsequent analysis we considered only the most probable 4 clusters for each site. Because of limits on the precision of force fields used in calculating the probabilities, we make no attempt to discriminate among representative structures for these clusters. Instead, we predict RDCs and PCSs for isotopically labeled glycan sites in each structure, using the magnetic susceptibility tensor derived from protein data, and examine the correlation with experimental data.

### Data Analysis

Predictions of PCS data and RDC data were made using the five independent Cartesian variables saved from the analysis module of ASSIGN_SLP and the coordinates for isotopically labeled sites (acetyl methyls and C1 carbons for glycan residues, alanine and valine methyls for protein residues). The back-calculation of protein data was used to confirm agreement with protein resonance assignments. The predicted glycan data are listed in Tables 1 and 2. Experimental data were entered in pairs of PCS and RDC data and placed adjacent to the best fit to a predicted set of data. The MATLAB app used to make predictions is included in Supporting Information. Depictions of PCS surfaces and molecular interactions between glycans and the protein surface were made with Chimera X.^37^

## Supplementary Material



## Data Availability

Coordinates for the structures discussed, CEACAM-Ig1 with the first two GlcNAc residues at site 104 (f28137 and f17916) have been deposited as a structure produced by “integrated and hybrid methods” with the temporary pdb accession code: 9AA9; The NMR assignments and associated data have been deposited with the BMRB accession code: 53402.

## Notes

MATLAB scripts and apps used to 1. Facilitate extraction of PCS data from a pair of HSQC spectra. 2. Predict RDC and PCS values given coordinates for the relevant atoms and a file (yy_file) containing the five independent Cartesian variables used to calculate a susceptibility tensor. 3. Transform initial protein coordinates to the principal alignment frame. 4. Calculate a set of points at a constant PCS value given a distance for the maximum extent and a yy_file. It also contains a detailed description of methods for the measurement of RDCs and a set of NMR data (Tm^3+^, Lu^3+^) used to determine crosspeak assignments and extract a yy_file. (HSQC chemical shifts, RDC-PCS pairs, PREs and NOEs). (PDF)
