# Supplementary material for: Conformational Preferences for N‑Glycans at the Surface of CEACAM1-Ig1
Source: ACS Chem Biol. 2025 Dec 19;21(1):130–40. doi: 10.1021/acschembio.5c00746 (PMC12813971; doi:10.1021/acschembio.5c00746)
Supplement: Supplementary file 1 [file cb5c00746_si_001.pdf]

Supporting Information

Conformational Preferences for N-glycans at the Surface of CEACAM1-Ig1

Alexander Eletsky, Chin Huang, Yinglong Miao, Kelley W Moremen, Laura C Morris and James H Prestegard*

Contents:

PCS_extract_f.mlapp – a MATLAB app to facilitate extraction of PCS data from a pair of HSQC or HETCOR spectra; one for a sample containing a diamagnetic ion and one for a sample containing a paramagnetic ion. Output is an EXCEL workbook with chemical shifts for connected crosspeaks.

RDC_PCS_BackCalc_e.mlapp – a MATLAB app to back-calculate RDC and PCS values for specific sites given coordinates for the relevant pairs of atoms and a file (yy_file) containing five independent cartesian variables provided by the RDC/PCS version of the ASSIGN_SLP program. Coordinates are taken from a modified prediction workbook used as an input for ASSIGN_SLP. Output is a pair of .mat files, one for RDCs and one for PCSs. These can be converted to text files by loading them into matlab saving them using the matlab command: save('filename','filename.mat','-ascii').

Principal_frame_transform_new5.m – a MATLAB script to transform coordinates, from the frame in which PCS and RDC were assigned to sites by the ASSIGN_SLP program, to the principal alignment frame. It uses the yy_file, containing five independent cartesian variables and saved by the analysis module of ASSIGN_SLP. It converts these to susceptibility tensor values, finds the eigenvalues of the tensor and uses the corresponding eigenvectors to transform the original coordinates. As RDCs and PCSs are insensitive to coordinate inversion, the structure returned can be a mirror image. This needs to be checked by examining the structure and corrected if necessary.

PCS_points_5.m – This script uses eta values from a diagonalized susceptibility tensor, and a selected distance R along the z axis to calculate a set of points (number of increments in theta and phi) at which the PCS value would be constant. The points can be scanned with a molecular display program to produce a surface of constant PCS value. The ppm value at this surface can be calculated using the formula: PCSmax*ChiAx*(2/3)/R^3. (R in Å, PCSmax 9.32e6, ChiAx saved by principal_frame-transform script).

Methods for RDC measurement – Details of a new NMR pulse sequence for the collection of RDCs using ^{13}C – detection are presented.

CEACAM_experimental Data –The HSQC sheet contains assigned shifts for a Lu^{3+} complex. The RDC-PCS 1 sheet contains RDCs and PCSs for a Tm^{3+} complex. The PRE sheet contains intensity data for a Gd^{3+} complex. The NOE sheets contain intensities and proton chemical shifts for NOE crosspeaks. These data were used to make crosspeak assignments and extract the independent Cartesian variables used in determination of a susceptibility tensor for the Tm^{3+} complex.

The MATLAB app for clustering trajectory frames and the MATLAB script that provides Z and d file input for the app were provided as a supplement to our previous publication.{Rogals, 2022 #35}

PCS_extract_f.mlapp

```
classdef PCS_extract_f < matlab.apps.AppBase

    % Properties that correspond to app components
    properties (Access = public)
        UIFigure                matlab.ui.Figure
        PickPeaksButton         matlab.ui.control.Button
        SaveExitButton          matlab.ui.control.Button
        Plot2Button             matlab.ui.control.Button
        FileName2EditField      matlab.ui.control.EditField
        FileName2Label          matlab.ui.control.Label
        Slider                  matlab.ui.control.Slider
        SliderLabel             matlab.ui.control.Label
        FileName1EditField      matlab.ui.control.EditField
        FileName1EditFieldLabel matlab.ui.control.Label
        Plot1Button             matlab.ui.control.Button
        UIAxes2                 matlab.ui.control.UIAxes
        UIAxes                  matlab.ui.control.UIAxes
    end

    properties (Access = private)
        FileName1 % Spectrum to load
        FileName2 % Spectrum to load
        Input1 % data to plot
        Input2 % data to plot
        startPt % slider start value
        ptNum
        x1
        y1
        x2
        y2
        ln1
        ln2
    end


    % Callbacks that handle component events
    methods (Access = private)

        % Button pushed function: Plot1Button
        function Plot1ButtonPushed(app, event)
```

```
        % Plot Figure1
        %Load a structured variable with fields image, ppm1 and ppm2.
        %The variable should be named Input1 and the file name should end with
_subSpec.mat
        % Omit the .mat extension when entering the name
        ax = app.UIAxes;
        datacursormode(ax,'on');
        ax.XDir = 'reverse'; ax.YDir = 'reverse';
        load(strcat(app.FileName1,'.mat'));
        app.Input1 = Input1;
        iptPointerManager(app.UIFigure);
        app.Slider.Limits = [min(app.Input1.ppm1-range(app.Input1.ppm2))
max(app.Input1.ppm1)];
        app.ptNum = 1;
        contour(app.Input1.ppm1, app.Input1.ppm2, app.Input1.image, 50, 'Parent',app.UIAxes);
        app.x1 = zeros(50,1);
        app.y1 = zeros(50,1);
        app.x2 = zeros(50,1);
        app.y2 = zeros(50,1);

    end

    % Callback function: FileName1EditField, UIAxes
    function FileNameEditFieldValueChanged(app, event)
        app.FileName1 = app.FileName1EditField.Value;

    end

    % Value changed function: Slider
    function SliderValueChanged(app, event)

        app.startPt = app.Slider.Value;
        delete(app.ln1);
        delete(app.ln2);
        xFnl = max(app.Input1.ppm1); xInl = min(app.Input1.ppm1);
        yFnl = max(app.Input1.ppm2); yInl = min(app.Input1.ppm2);
        Rngx = range(app.Input1.ppm1); Rngy = range(app.Input1.ppm2);
        % plot a new line of slope 1
        app.ln1 = line(app.UIAxes, [max(app.startPt, xInl) min(Rngy+app.startPt, xFnl)],
[max(yInl,(yInl+xInl-app.startPt)) min((yInl+xFnl-app.startPt),yFnl)], 'Color', 'r');
        app.ln2 = line(app.UIAxes2, [max(app.startPt,xInl) min(Rngy+app.startPt, xFnl)],
[max(yInl,(yInl+xInl-app.startPt)) min(yInl+xFnl-app.startPt, yFnl)], 'Color', 'r');

    end

    % Value changed function: FileName2EditField
```

```
        function FileName2EditFieldValueChanged(app, event)
            app.FileName2 = app.FileName2EditField.Value;
        end

        % Button pushed function: Plot2Button
        function Plot2ButtonPushed(app, event)
            % Plot Figure 2
            %The variable should be named Input2 and the file name should end with
_subSpec.mat
            % Omit the .mat extension when entering the name
            ax2 = app.UIAxes2;
            ax2.XDir = 'reverse'; ax2.YDir = 'reverse';
            load(strcat(app.FileName2,'.mat'));
            app.Input2 = Input1;
            contour(app.Input2.ppm1, app.Input2.ppm2, app.Input2.image, 50,
'Parent',app.UIAxes2)

        end

        % Button pushed function: SaveExitButton
        function SaveExitButtonPushed(app, event)
            xy = [app.x1(1:app.ptNum),app.y1(1:app.ptNum),app.x2(1:app.ptNum),
app.y2(1:app.ptNum)];
            %xy_nz = nonzeros(xy);
            %xy_reshape = reshape(xy_nz,(app.ptNum-1),[]);
            delete(app.ln1);
            delete(app.ln2);
            %save(strcat(app.FileName1,'_xy_points.mat'),'xy');
            writematrix(xy,strcat(app.FileName2,'.xlsx'));
            exportgraphics(app.UIAxes,strcat(app.FileName1,'.jpg'));
            exportgraphics(app.UIAxes2,strcat(app.FileName2,'.jpg'));
            return;
        end

        % Button pushed function: PickPeaksButton
        function PickPeaksButtonPushed2(app, event)
            % Pick points;
            app.UIFigure.HandleVisibility = 'callback';
                [app.x1(app.ptNum),app.y1(app.ptNum)] = ginput(1);
                [app.x2(app.ptNum),app.y2(app.ptNum)] = ginput(1);
            app.UIFigure.HandleVisibility = 'on';

text(app.x1(app.ptNum),app.y1(app.ptNum),num2str(app.ptNum),'Color','r','VerticalAlignment','t
op','Parent',app.UIAxes);

text(app.x2(app.ptNum),app.y2(app.ptNum),num2str(app.ptNum),'Color','r','VerticalAlignment','t
op','Parent',app.UIAxes2);
```

```
            app.ptNum = app.ptNum+1;

        end
    end

    % Component initialization
    methods (Access = private)

        % Create UIFigure and components
        function createComponents(app)

            % Create UIFigure and hide until all components are created
            app.UIFigure = uifigure('Visible', 'off');
            app.UIFigure.Position = [100 100 858 480];
            app.UIFigure.Name = 'MATLAB App';

            % Create UIAxes
            app.UIAxes = uiaxes(app.UIFigure);
            title(app.UIAxes, 'Plot 1')
            xlabel(app.UIAxes, 'X')
            ylabel(app.UIAxes, 'Y')
            zlabel(app.UIAxes, 'Z')
            app.UIAxes.ButtonDownFcn = createCallbackFcn(app,
@FileNameEditFieldValueChanged, true);
            app.UIAxes.Position = [12 10 408 384];

            % Create UIAxes2
            app.UIAxes2 = uiaxes(app.UIFigure);
            title(app.UIAxes2, 'Plot 2')
            xlabel(app.UIAxes2, 'X')
            ylabel(app.UIAxes2, 'Y')
            zlabel(app.UIAxes2, 'Z')
            app.UIAxes2.Position = [427 10 413 384];

            % Create Plot1Button
            app.Plot1Button = uibutton(app.UIFigure, 'push');
            app.Plot1Button.ButtonPushedFcn = createCallbackFcn(app, @Plot1ButtonPushed,
true);
            app.Plot1Button.Position = [293 435 57 23];
            app.Plot1Button.Text = 'Plot 1';

            % Create FileName1EditFieldLabel
            app.FileName1EditFieldLabel = uilabel(app.UIFigure);
            app.FileName1EditFieldLabel.HorizontalAlignment = 'right';
            app.FileName1EditFieldLabel.Position = [20 436 70 22];
            app.FileName1EditFieldLabel.Text = 'File Name 1';
```

```
            % Create FileName1EditField
            app.FileName1EditField = uieditfield(app.UIFigure, 'text');
            app.FileName1EditField.ValueChangedFcn = createCallbackFcn(app,
@FileNameEditFieldValueChanged, true);
            app.FileName1EditField.Position = [105 436 176 22];

            % Create SliderLabel
            app.SliderLabel = uilabel(app.UIFigure);
            app.SliderLabel.HorizontalAlignment = 'right';
            app.SliderLabel.Position = [363 436 36 22];
            app.SliderLabel.Text = 'Slider';

            % Create Slider
            app.Slider = uislider(app.UIFigure);
            app.Slider.Limits = [16 24];
            app.Slider.ValueChangedFcn = createCallbackFcn(app, @SliderValueChanged, true);
            app.Slider.Position = [420 445 316 3];
            app.Slider.Value = 16;

            % Create FileName2Label
            app.FileName2Label = uilabel(app.UIFigure);
            app.FileName2Label.HorizontalAlignment = 'right';
            app.FileName2Label.Position = [20 405 70 22];
            app.FileName2Label.Text = 'File Name 2';

            % Create FileName2EditField
            app.FileName2EditField = uieditfield(app.UIFigure, 'text');
            app.FileName2EditField.ValueChangedFcn = createCallbackFcn(app,
@FileName2EditFieldValueChanged, true);
            app.FileName2EditField.Position = [105 405 176 22];

            % Create Plot2Button
            app.Plot2Button = uibutton(app.UIFigure, 'push');
            app.Plot2Button.ButtonPushedFcn = createCallbackFcn(app, @Plot2ButtonPushed,
true);
            app.Plot2Button.Position = [293 405 57 23];
            app.Plot2Button.Text = 'Plot 2';

            % Create SaveExitButton
            app.SaveExitButton = uibutton(app.UIFigure, 'push');
            app.SaveExitButton.ButtonPushedFcn = createCallbackFcn(app,
@SaveExitButtonPushed, true);
            app.SaveExitButton.Position = [758 405 82 23];
            app.SaveExitButton.Text = 'Save & Exit';

            % Create PickPeaksButton
            app.PickPeaksButton = uibutton(app.UIFigure, 'push');
```

```
            app.PickPeaksButton.ButtonPushedFcn = createCallbackFcn(app,
@PickPeaksButtonPushed2, true);
            app.PickPeaksButton.Position = [758 435 82 23];
            app.PickPeaksButton.Text = 'Pick Peaks';

            % Show the figure after all components are created
            app.UIFigure.Visible = 'on';
        end
    end

    % App creation and deletion
    methods (Access = public)

        % Construct app
        function app = PCS_extract_f

            % Create UIFigure and components
            createComponents(app)

            % Register the app with App Designer
            registerApp(app, app.UIFigure)

            if nargout == 0
                clear app
            end
        end

        % Code that executes before app deletion
        function delete(app)

            % Delete UIFigure when app is deleted
            delete(app.UIFigure)
        end
    end
end
```

RDC_PCS_BackCalc_e.mlapp

```
classdef RDC_PCS_BackCalc_e < matlab.apps.AppBase

    % Properties that correspond to app components
    properties (Access = public)
        UIFigure                  matlab.ui.Figure
        PlotRs                   matlab.ui.control.Button
        PlotRDCsButton              matlab.ui.control.Button
        UITable                  matlab.ui.control.Table
        ReadButton_3               matlab.ui.control.Button
        ExcelExprFileNameEditField   matlab.ui.control.EditField
        ExcelExprFileNameEditFieldLabel  matlab.ui.control.Label
        CalculateButton             matlab.ui.control.Button
        ReadButton_2               matlab.ui.control.Button
        OrderParameterFileEditField  matlab.ui.control.EditField
        OrderParameterFileEditFieldLabel  matlab.ui.control.Label
        ReadButton                 matlab.ui.control.Button
        ExcelPredFileNameEditField   matlab.ui.control.EditField
        ExcelPredFileNameEditFieldLabel  matlab.ui.control.Label
        RDCPCSBackCalcLabel         matlab.ui.control.Label
        UIAxes                   matlab.ui.control.UIAxes
    end

    % This version uses order parameters in back-calculation
    % JHP 06/03/25
    % Experiment files (at least RDCPCS) must be ordered by assignment if
    % experimental values and predictions are to be compared
    properties (Access = private)
        filename % Prediction Excel file name
        filenameExp % Experiment Excel file name
        OP % order parameters from prior search
        yy
        coords
        PCScoords
        back_calc_RDC
        back_calc_PCS
        Dmax
        RDCs
        PCSs
        PCSmax
        Data
        assign
        assign_new
        orderpar
    end
```

```
% Callbacks that handle component events
methods (Access = private)

    % Button pushed function: ReadButton
    function ReadButtonPushed(app, event)
        RDCcoords = readtable(app.filename,"Sheet",'Coordinates & Order Parameters');
        app.coords = table2array(RDCcoords(:,2:7));
        app.Dmax = table2array(RDCcoords(:,8));
        app.orderpar = table2array(RDCcoords(:,9)); % modified 06/03/25 to add orderpar
        app.Dmax = app.Dmax.*app.orderpar;
        app.PCScoords = readtable(app.filename,"Sheet",'PCS');
        app.PCSmax = table2array(app.PCScoords(:,8));
        app.PCScoords = table2array(app.PCScoords(:,2:7));

    end

    % Button pushed function: ReadButton_2
    function ReadButton_2Pushed(app, event)
        yyy = load(app.OP);
        app.yy = yyy.yy;
    end

    % Button pushed function: CalculateButton
    function CalculateButtonPushed(app, event)
        % BackCalculate RDCs
        numData = size(app.coords,1);
        x = app.coords(:,1) - app.coords(:,4);
        y = app.coords(:,2) - app.coords(:,5);
        z = app.coords(:,3) - app.coords(:,6);

        a = zeros(numData,5);

        % generate matrix of independent spatial terms
        a(:,1) = (y.^2 - x.^2)';
        a(:,2) = (z.^2 - x.^2)';
        a(:,3) = 2*(x.*y)';
        a(:,4) = 2*(x.*z)';
        a(:,5) = 2*(y.*z)';
        a = app.Dmax .* a .*vecnorm([x, y, z]')'.^-5;

        app.back_calc_RDC = a * app.yy;

        % BackCalculate PCSs
        x = app.PCScoords(:,1) - app.PCScoords(:,4);
        y = app.PCScoords(:,2) - app.PCScoords(:,5);
        z = app.PCScoords(:,3) - app.PCScoords(:,6);
```

```
        a = zeros(numData,5);

        % generate matrix of independent spatial terms
        a(:,1) = (y.^2 - x.^2)';
        a(:,2) = (z.^2 - x.^2)';
        a(:,3) = 2*(x.*y)';
        a(:,4) = 2*(x.*z)';
        a(:,5) = 2*(y.*z)';
        a = app.PCSmax .* a .*vecnorm([x, y, z]')'.^-5;

        app.back_calc_PCS = a * app.yy;

        % create a table to present data

        app.Data = [app.RDCs, app.back_calc_RDC, app.PCSs, app.back_calc_PCS,
app.assign_new];
        app.UITable.ColumnName = {'RDCexp', 'RDCcalc', 'PCS_exp', 'PCScalc','Assign'};
        app.UITable.FontSize = 12;
        app.UITable.Data = app.Data;

        % save RDCcalc and PCScalc
        tempRDC = app.Data(:,2);
        save('BackCalc_RDC','tempRDC');
        tempPCS = app.Data(:,4);
        save('BackCalc_PCS','tempPCS');

    end

    % Value changed function: ExcelPredFileNameEditField
    function ExcelPredFileNameEditFieldValueChanged(app, event)
        app.filename = app.ExcelPredFileNameEditField.Value;

    end

    % Value changing function: OrderParameterFileEditField
    function OrderParameterFileEditFieldValueChanging(app, event)
        app.OP = event.Value;

    end

    % Value changed function: ExcelExprFileNameEditField
    function ExcelExprFileNameEditFieldValueChanged(app, event)
        app.filenameExp = app.ExcelExprFileNameEditField.Value;
    end

    % Button pushed function: ReadButton_3
```

```
function ReadButton_3Pushed(app, event)
   app.RDCs = readmatrix(app.filenameExp,"Sheet",'RDC-PCS 1','Range','B:B');
   app.RDCs = app.RDCs(2:end);
   app.PCSs = readmatrix(app.filenameExp,"Sheet",'RDC-PCS 1','Range','D:D');
   app.PCSs = app.PCSs(2:end);
   app.assign = readcell(app.filenameExp,"Sheet",'RDC-PCS 1','Range','A:A');
   app.assign = app.assign(2:end);
   app.assign_new = readmatrix(app.filenameExp,"Sheet",'RDC-PCS 1','Range','F:F');
   app.assign_new = floor(app.assign_new(2:end));
   app.UITable.RowName = app.assign;
end

% Button pushed function: PlotRDCsButton
function PlotRDCsButtonPushed(app, event)
   % the folowing revised from Williams analysis.app

   notMissing = app.RDCs ~= 999;
   RDCexp = app.RDCs(notMissing);
   RDCcalc = app.back_calc_RDC(notMissing);

   m = max(abs([RDCexp;RDCcalc]));
   x = linspace(-m,m);
   y = x;

   plot(app.UIAxes,RDCexp,RDCcalc,'o',x,y,'--');
   R = corrcoef(RDCexp,RDCcalc,'Rows','complete');
   plotTitle = sprintf('R = %.2f',R(1,2));
   app.UIAxes.Title.String = plotTitle;
   app.UIAxes.XLabel.String = 'Experiment';
   app.UIAxes.YLabel.String = 'Calculated';
end

% Button pushed function: PlotRs
function PlotRsButtonPushed(app, event)
   % the folowing revised from Williams analysis.app

   notMissing = app.PCSs ~= 999;
   PCSexp = app.PCSs(notMissing);
   PCScalc = app.back_calc_PCS(notMissing);

   m = max(abs([PCSexp;PCScalc]));
   x = linspace(-m,m);
   y = x;

   plot(app.UIAxes,PCSexp,PCScalc,'o',x,y,'--');
   R = corrcoef(PCSexp,PCScalc,'Rows','complete');
   plotTitle = sprintf('R = %.2f',R(1,2));
```

```
            app.UIAxes.Title.String = plotTitle;
            app.UIAxes.XLabel.String = 'Experiment';
        end
    end

    % Component initialization
    methods (Access = private)

        % Create UIFigure and components
        function createComponents(app)

            % Create UIFigure and hide until all components are created
            app.UIFigure = uifigure('Visible', 'off');
            app.UIFigure.Position = [100 100 640 480];
            app.UIFigure.Name = 'MATLAB App';

            % Create UIAxes
            app.UIAxes = uiaxes(app.UIFigure);
            title(app.UIAxes, 'Title')
            xlabel(app.UIAxes, 'X')
            ylabel(app.UIAxes, 'Y')
            zlabel(app.UIAxes, 'Z')
            app.UIAxes.Position = [319 14 310 291];

            % Create RDCPCSBackCalcLabel
            app.RDCPCSBackCalcLabel = uilabel(app.UIFigure);
            app.RDCPCSBackCalcLabel.Position = [263 440 116 22];
            app.RDCPCSBackCalcLabel.Text = 'RDC PCS Back Calc';

            % Create ExcelPredFileNameEditFieldLabel
            app.ExcelPredFileNameEditFieldLabel = uilabel(app.UIFigure);
            app.ExcelPredFileNameEditFieldLabel.HorizontalAlignment = 'right';
            app.ExcelPredFileNameEditFieldLabel.Position = [88 401 121 22];
            app.ExcelPredFileNameEditFieldLabel.Text = 'Excel Pred File Name';

            % Create ExcelPredFileNameEditField
            app.ExcelPredFileNameEditField = uieditfield(app.UIFigure, 'text');
            app.ExcelPredFileNameEditField.ValueChangedFcn = createCallbackFcn(app,
@ExcelPredFileNameEditFieldValueChanged, true);
            app.ExcelPredFileNameEditField.Position = [224 401 163 22];

            % Create ReadButton
            app.ReadButton = uibutton(app.UIFigure, 'push');
            app.ReadButton.ButtonPushedFcn = createCallbackFcn(app, @ReadButtonPushed,
true);
            app.ReadButton.Position = [397 401 52 23];
            app.ReadButton.Text = 'Read';
```

```
        % Create OrderParameterFileEditFieldLabel
        app.OrderParameterFileEditFieldLabel = uilabel(app.UIFigure);
        app.OrderParameterFileEditFieldLabel.HorizontalAlignment = 'right';
        app.OrderParameterFileEditFieldLabel.Position = [90 318 118 22];
        app.OrderParameterFileEditFieldLabel.Text = 'Order Parameter File';

        % Create OrderParameterFileEditField
        app.OrderParameterFileEditField = uieditfield(app.UIFigure, 'text');
        app.OrderParameterFileEditField.ValueChangingFcn = createCallbackFcn(app,
@OrderParameterFileEditFieldValueChanging, true);
        app.OrderParameterFileEditField.Position = [223 318 163 22];

        % Create ReadButton_2
        app.ReadButton_2 = uibutton(app.UIFigure, 'push');
        app.ReadButton_2.ButtonPushedFcn = createCallbackFcn(app,
@ReadButton_2Pushed, true);
        app.ReadButton_2.Position = [397 318 52 23];
        app.ReadButton_2.Text = 'Read';

        % Create CalculateButton
        app.CalculateButton = uibutton(app.UIFigure, 'push');
        app.CalculateButton.ButtonPushedFcn = createCallbackFcn(app,
@CalculateButtonPushed, true);
        app.CalculateButton.Position = [507 401 65 23];
        app.CalculateButton.Text = 'Calculate';

        % Create ExcelExprFileNameEditFieldLabel
        app.ExcelExprFileNameEditFieldLabel = uilabel(app.UIFigure);
        app.ExcelExprFileNameEditFieldLabel.HorizontalAlignment = 'right';
        app.ExcelExprFileNameEditFieldLabel.Position = [88 361 120 22];
        app.ExcelExprFileNameEditFieldLabel.Text = 'Excel Expr File Name';

        % Create ExcelExprFileNameEditField
        app.ExcelExprFileNameEditField = uieditfield(app.UIFigure, 'text');
        app.ExcelExprFileNameEditField.ValueChangedFcn = createCallbackFcn(app,
@ExcelExprFileNameEditFieldValueChanged, true);
        app.ExcelExprFileNameEditField.Position = [223 361 166 22];

        % Create ReadButton_3
        app.ReadButton_3 = uibutton(app.UIFigure, 'push');
        app.ReadButton_3.ButtonPushedFcn = createCallbackFcn(app,
@ReadButton_3Pushed, true);
        app.ReadButton_3.Position = [397 361 52 23];
        app.ReadButton_3.Text = 'Read';

        % Create UITable
```

```
            app.UITable = uitable(app.UIFigure);
            app.UITable.ColumnName = {'Assign'; 'RDCcalc'; 'RDCexp'; 'PCScalc'};
            app.UITable.RowName = {};
            app.UITable.Position = [18 31 302 263];

            % Create PlotRDCsButton
            app.PlotRDCsButton = uibutton(app.UIFigure, 'push');
            app.PlotRDCsButton.ButtonPushedFcn = createCallbackFcn(app,
@PlotRDCsButtonPushed, true);
            app.PlotRDCsButton.Position = [499 361 100 23];
            app.PlotRDCsButton.Text = 'Plot RDCs';

            % Create PlotRs
            app.PlotRs = uibutton(app.UIFigure, 'push');
            app.PlotRs.ButtonPushedFcn = createCallbackFcn(app, @PlotRsButtonPushed, true);
            app.PlotRs.Position = [499 317 100 23];
            app.PlotRs.Text = 'Plot PSCs';

            % Show the figure after all components are created
            app.UIFigure.Visible = 'on';
        end
    end

    % App creation and deletion
    methods (Access = public)

        % Construct app
        function app = RDC_PCS_BackCalc_e

            % Create UIFigure and components
            createComponents(app)

            % Register the app with App Designer
            registerApp(app, app.UIFigure)

            if nargout == 0
                clear app
            end
        end

        % Code that executes before app deletion
        function delete(app)
            % Delete UIFigure when app is deleted
            delete(app.UIFigure)
        end
    end
end
```

Principal_frame_transform_new5.m

```
%% Script to put a pdb file in the principal frame of a Chi tensor
% starting from a cartesian yy file and putting
%  Ln3+ at 0,0,0. Output is the pdb file =, ChiAx, ChiRh and Eta.
% revised 01/11/25, revised 04/26/25 to have z inverse option
% revised 05/15/25 to use new Chi definitions checked 5/29/25
% revised 06/10/25 to reorder D and V
%%
% read the cartesian delta chi file (yy_file.mat);
load('yy_file_Tm_3p88.mat'); % use name of yy_file saved by ASSIGN_SLP
% make a delta chi tensor - modified to use zyx vs xyz yy values
chi = zeros(3); % from Bertini 2002 & Ubbink 2022
chi(1,1) = -yy(2)-yy(1); chi(2,2) = yy(1); chi(3,3) = yy(2);
chi(1,2) = yy(3); chi(2,1) = yy(3);
chi(1,3) = yy(4); chi(3,1) = yy(4);
chi(2,3) = yy(5); chi(3,2) = yy(5);

[V,D] = eig(chi);
ChiAx = D(3,3)-(D(1,1)+D(2,2))/2;
ChiRh = D(1,1)-D(2,2);
%% Reorder D and V to make D(33) largest magnitude
if abs(D(3,3)) < abs(D(1,1))
  Dtemp = D(1,1); D(1,1) = D(3,3);
  D(3,3) = Dtemp;
  V = V([1 2 3],[3 2 1]);
end
Eta = (D(1,1)-D(2,2))/D(3,3); % points script needs to use this definition
% this goes from 0 to 1 and differs from Ubbink 2022 definition
%% Transform pdb file to principal frame. Give file name of original file
filename = 'CEACAM_L4_AlphaF_GaMD_f295131_TbMov_trny90';
pdbfile = cat(2,filename,'.pdb');
% read the file using Matsunaga's readpdb function - must be on path
% ymatsunaga-mdtoolbox-2f57397
pdb = readpdb(pdbfile);
sizeCoords = size(pdb.xyz,1);
%% Transform coordinates, find 'Tb, center Tb
pdb.xyz = (V'*pdb.xyz')';
% Zero Tb
logic = (string(pdb.name) == "TB  ");
line_num = find(logic);
TbCoords = pdb.xyz(line_num,:);
for ii = 1:sizeCoords
   pdb.xyz(ii,:) = pdb.xyz(ii,:)-TbCoords;
   %pdb.xyz(ii,1) = -pdb.xyz(ii,1); % make mirror image
end
%%
```

```
outfile = cat(2,filename,'_ChiAxChiRh.mat'); % initial pdb file name with extension
save(outfile,'ChiAx','ChiRh','Eta');
outfile = cat(2,filename,'_paf_3p88.pdb'); % initial pdb filename with extnsion
writepdb(outfile, pdb);
```

PCS_points_5.m

```
% program to generate points at a constant PCS value
out_file = fopen('PCS_50points_R40_Eta-0p45.pdb','w');
formatSpec1 = 'ATOM %6d  C1  %3s A 500    %7.3f %7.3f %7.3f  1.00 10.00           C\n';
%formatSpec1 = 'ATOM   %4d %3s  PCS   500     %7.3f %7.3f %7.3f  1.00 10.00      C \n';
% number of points in theta and phi
Num = 50;
ThetaInc = pi/Num;
PhiInc0 = 2*pi/Num;
% enter susceptibility tensor as max distance and eta
Eta = -0.45; % (D22-D11)/D33 runs from 0 to 1
Rmax = 40;
Rmax = Rmax/2^0.333; % at theta = 0 angular factor is 2 - raised to the 1/3 power
% enter coordinates of paramagnetic ion
X_off = 0.0;
Y_off = 0.0;
Z_off = 0.0;
% give beginning atom number for pdb file
Atom_start = 1;
for i = 1:Num-1
   Theta = i*ThetaInc;
   for j = 1:ceil(Num*sin(Theta))
      PhiInc = PhiInc0/sin(Theta);
      Atom = Atom_start+j+(i-1)*Num;
      Phi = j*PhiInc;
      Angle = 3*cos(Theta)^2-1+Eta*sin(Theta)^2*cos(2*Phi); % changed - no 1/2
      if Theta < pi/2
         type = 'PPS';
      else
         type = 'PNS';
      end
      if Angle < 0.0
         type = 'NEG';
      end
      r = Rmax*abs(Angle^(1/3)); % Rmax = ChiAx*2/(PCS*12+pi)
      % PCS = PCSmax*ChiAx/(6*pi*Rmax^3) % at theta = 0
      x = r*sin(Theta)*cos(Phi)+X_off; y = r*sin(Theta)*sin(Phi)+Y_off; z = r*cos(Theta)+Z_off;
      fprintf(out_file,formatSpec1,Atom,type,x,y,z);
   end
end
fclose all;
```

Methods for RDC measurement using ^{13}C – detection

For RDC measurement, pseudo-3D J-modulated ^{13}C HETCOR spectra were collected for Lu^{3+}, Tb^{3+} and Tm^{3+} complexes. An improved version (Figure 7) of a previously used J-modulation pulse sequence[25] was employed that included a single constant-time (CT) period for both J-modulation and ^{1}H frequency labeling, as well as adiabatic ^{13}C inversion and refocusing pulses. The refocusing pulse was a composite 20% smoothed CHIRP pulse with 80 kHz sweep and 2000 us pulse width. Recycle delay, steady-state scans, scans per increment, spectral widths, frequency offsets, and ^{13}C time-domain points were the same as for 2D SCT-HETCOR. Total constant-time delay was 2T was set to 16 ms, limiting ^{1}H time-domain complex points to 109. A total of 12 J-modulation evolution delays 2τ were sampled at 0.6, 1.8, 3.0, 4.4, 5.8, 7.2, 8.4, 9.6, 11.0, 13.2, 14.4, and 15.4 ms. Total acquisition times per spectrum were 13 h (Lu^{3+} and Tm^{3+} samples) and 32 h (Tb^{3+} sample). All SCT-HETCOR and J-modulated CT-HETCOR spectra were Fourier-transformed using NMRPipe.

Peaks in 2D ^{1}H-^{13}C planes of J-modulated ^{13}C CT-HETCOR were integrated over elliptical regions using NMRViewJ. ^{1}H-^{13}C coupling values and their standard error estimates were obtained via non-linear least squares (NLLS) fit of a three-parameter sinusoid function (Eq 1S) to peak integral series data in MATLAB. Here the variable parameters are amplitude A, coupling K, and time correction τ_0. The τ_0 parameter was used to account for variation of effective coupling evolution times of different signals, depending on the ^{13}C shift offset and the finite length of the ^{13}C adiabatic inversion pulse. For diamagnetic Lu^{3+} complex the apparent ^{1}H-^{13}C coupling K corresponds to the scalar coupling, K(Lu) = $^{1}J_{CH}$, while for paramagnetic complexes it corresponds to a sum of scalar and residual dipolar couplings, K(Tb) = $^{1}J_{CH}$ + $^{1}D_{CH}$(Tb), and K(Tm) = $^{1}J_{CH}$ + $^{1}D_{CH}$(Tm). Since the inherent variation of $^{1}J_{CH}$ scalar couplings within two groups of resonance corresponding to different atomic linkage types (Ala/Val and GlcNAc methyl vs. GlcNAc C1) is lower than NLLS fit errors, the averaged coupling values and their standard errors were calculated within each group for Lu^{3+} complex data and were used for subsequent calculations. RDCs were thus computed as the difference between apparent couplings for anisotropic (Tb^{3+}, Tm^{3+}) and isotropic (Lu^{3+}) complexes, $^{1}D_{CH}$(Tb) = K(Tb) – K(Lu) and $^{1}D_{CH}$(Tm) = K(Tm) – K(Lu), with standard errors determined according to error propagation.

$$V(\tau) = Asin\big(2\pi K(\tau - \tau_0)\big) \qquad \text{Eq 1S}$$

An example of J-modulated data and fits to this equation were given in Figure 4 of the manuscript.

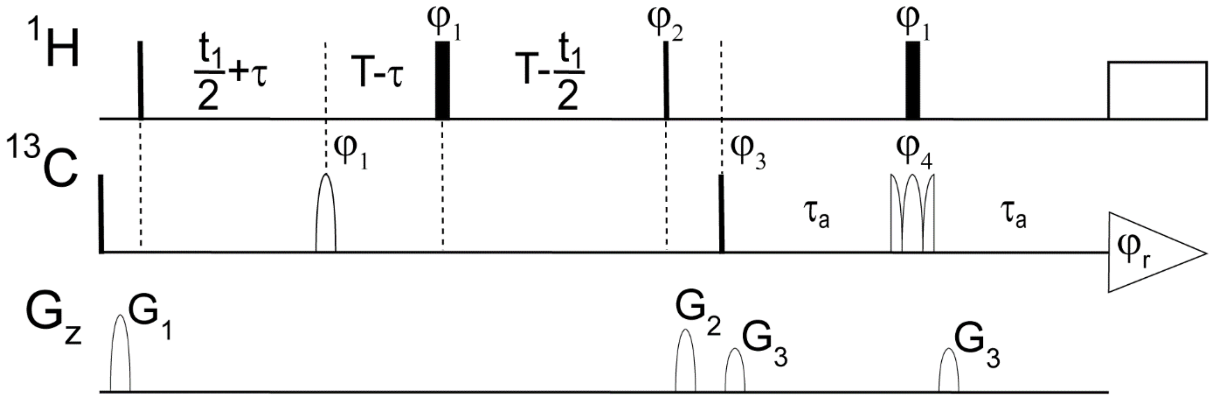

Figure 1S. Pulse sequence diagram of J-modulated pseudo-3D CT-HETCOR experiment for measuring ^{1}H-^{13}C RDCs. Narrow and wide rectangles represent 90° and 180° high power pulses, respectively. One- and three-lobed elements represent ^{13}C adiabatic inversion and refocusing pulses, respectively. Delay τ_a is set to 1.0 ms as a compromise value for optimal polarization transfer for both methyl and C1 sites. J-modulation via ^{1}H-^{13}C couplings is produced by variation of the τ delay, while constant-time implementation avoids unwanted modulations from ^{1}H-^{1}H couplings. The length of the constant time delay 2T (16 ms) is chosen to balance the acceptable resolution in the ^{1}H dimension and precision of RDC measurement on the one hand, and the signal decay due to relaxation losses, on the other. Gradient pulses are G1 (1.0 ms, 26.5 G/cm), G2 (1.0 ms, 15.9 G/cm), G3 (0.6 ms, 9.0 G/cm). Phases were φ_1 = {x, x, -x, x-}, φ_2 = {y, -y}, φ_3 = {4(x), 4(y), 4(-x), 4(-y)}, φ_4 = {2(x), 2(y), 2(-x), 2(-y)}, φ_r = {x, -x, x, -x, y, -y, y, -y, -x, x, -x, x, -y, y, -y, y}. Quadrature detection in the indirect ^{1}H dimension is achieved by incrementing the φ_2 pulse by 90°.

Experimental data used in Susceptibility tensor determination

HSQC data

Sample Name CEACAM
Experiment "1H,15N-HSQC"
Instrument 600 13C
Date 3/5/2022
Comments added 0.3 to 13C shifts 06/05/24
corrected to Lu3+ shifts (NOEs) 02/08/22

Assignment			w1	w2
A5	Me	19.2	1.33	
A2	Me	18.8	1.09	
A4	Me	22.5	1.26	
A7	Me	20.3	1.36	
A6	Me	22.2	1.26	

A3	Me	20.4	1.41
A8	Me	18.4	1.17
A9	Me	17	0.84
A1	Me	23.2	1.26
V16	Me	21.4	0.94
V14	Me	22	0.65
V11	Me	21.4	0.18
V10	Me	18.5	-0.32
V20	Me	22.6	0.62
V21	Me	23.6	1
V13	Me	21.2	0.51
V12	Me	19.65	0.31
V23	Me	22	0.95
V26	Me	21.5	1
V15	Me	21.7	0.63
V17	Me	20.8	0.6
V22	Me	22.1	0.86
V27	Me	20.4	0.95
V25	Me	21.4	0.99
V24	Me	22.2	0.88
V18	Me	21.2	0.67
V19	Me	22.3	0.71

PRE data

Sample Gd^{3+} intensities normalized to no Gd

Pulse Sequence HSQC
Instrument 600 MHz
Date new intensities 03/05/22

Peak	Intensity	Error	Amino Acid	assignnent
A5	1	0.2	A	5
A2	1.1	0.2	A	17
A4	0	0.2	A	54
A7	0	0.2	A	56
A6	0	0.2	A	63
A3	0.5	0.2	A	72
A8	1.1	0.2	A	77
A9	0.7	0.2	A	88
A1	1.1	0.2	A	117
V16	0.6	0.2	V	16.1
V14	1	0.2	V	16.2
V11	1	0.2	V	22.1
V10	1	0.2	V	22.2
V20	0.7	0.2	V	26.1
V21	0.8	0.2	V	26.2
V13	0.8	0.2	V	44.1
V12	0.8	0.2	V	44.2
V23	0.9	0.2	V	51.1
V26	0.86	0.2	V	51.2
V15	1	0.2	V	95.1
V17	0.8	0.2	V	95.2
V22	0.4	0.2	V	107.1
V27	0.82	0.2	V	107.2
V25	1.2	0.2	V	113.1
V24	1.1	0.2	V	113.2
V18	1	0.2	V	123.1
V19	1	0.2	V	123.2

RDC-PCS data – Tm^{3+}

Peak #	RDC	Error	PCS	Error	RDC/5	Amino	A	assignment
A5	999	999	999	999		A	5	
A2	-1.4	0.66	0.0801	0.02		A	17	
A4	999	999	999	999		A	54	
A7	999	999	999	999		A	56	
A6	999	999	999	999		A	63	
A3	999	999	1.5402	0.15		A	72	
A8	999	999	0.4706	0.0470		A	77	
A9	999	999	0.9334	0.0933		A	88	
A1	-0.62	0.52	0.3439	0.034		A	117	
V16	999	999	0.13	0.02		V	16.1	
V14	999	999	0.13	0.02		V	16.2	
V11	-0.6132		0.1856	0.18	0.02		V	22.1
V10	-0.2884		0.1865	0.205	0.0205		V	22.2
V20	-0.2310		0.5199	0.4322	0.0432		V	26.1
V21	-0.1212		0.3944	0.4626	0.0462		V	26.2
V13	999	999	0.6105	0.0610		V	44.1	
V12	999	999	999	999		V	44.2	
V23	999	999	1.08	0.11		V	51.1	
V26	999	999	1.16	0.12		V	51.2	
V15	999	999	0.16	0.02		V	95.1	
V17	-1.38	0.44	0.14	0.02		V	95.2	
V22	999	999	0.92	0.09		V	107.1	
V27	999	999	999	999		V	107.2	
V25	0.1984	0.1696	0	0.02		V	113.1	
V24	-0.5434		0.3421	-0.2	0.02		V	113.2
V18	-0.3362		0.3000	0.1448	0.02		V	123.1
V19	1.72468		0.3019	0.1776	0.02		V	123.2

NOE data

Sample Name CEACAM_L4_Val_Uglc Lu^{3+}
Experiment 3D 1H-15N NOESY

peak #	w1	w2	w3	Data Height	Amino Acid	assignment
A5	0.32	23.208	1.261	6530000	A	5
	0.613	23.178	1.262	14730000		
	1.254	23.184	1.26	301100000		
	1.724	23.2	1.264	5648000		
	4.792	23.181	1.26	34790000		
	8.775	23.187	1.262	12840000		
	8.984	23.208	1.259	6416000		
A2	0.641	18.788	1.093	9300000	A	17
	1.085	18.793	1.091	365700000		
	1.481	18.793	1.091	10790000		
	1.668	18.793	1.091	6843000		
	2.056	18.793	1.091	5336000		
	3.387	18.793	1.091	29450000		
	4.231	18.793	1.091	8711000		
	6.599	18.79	1.09	6337000		
	8.165	18.8	1.091	30730000		
A4	0.989	20.399	1.412	16380000	A	54
	1.405	20.399	1.412	344000000		
	4.65	20.391	1.412	5936000		
	5.024	20.389	1.41	4682000		
	5.436	20.392	1.413	17390000		
	6.447	20.395	1.412	14640000		
	8.976	20.41	1.414	6033000		
	9.304	20.389	1.414	6753000		
	0.921	20.399	1.412	10460000		
A7	1.258	22.464	1.264	471300000	A	56
	4.575	22.463	1.264	24940000		
	7.841	22.46	1.264	7221000		
A6	1.32	19.197	1.326	1698000000	A	63
	4.381	19.208	1.326	25760000		
	7.065	19.208	1.324	7138000		
	7.398	19.204	1.328	6518000		
	8.281	19.205	1.33	6410000		
A3	1.253	22.177	1.257	545400000	A	72
	4.513	22.172	1.256	27680000		
	7.681	22.18	1.256	8722000		

	7.936	22.169	1.256	11290000		
A8	1.352	20.284	1.357	575800000	A	77
	4.648	20.284	1.357	23330000		
	5.023	20.295	1.356	5027000		
	8.121	20.267	1.358	12370000		
A9	1.168	18.415	1.172	262000000	A	88
	1.657	18.397	1.17	6700000		
	1.957	18.395	1.17	6330000		
	3.677	18.423	1.174	6758000		
	4.618	18.424	1.172	30160000		
	8.133	18.434	1.169	12930000		
A1	0.318	17.023	0.838	5958000	A	117
	0.627	17.018	0.839	23380000		
	3.645	17.014	0.837	12270000		
	0.837	17.01	0.839	161000000		
V16	-0.321	18.452	-0.319	656900000	V	16.1
	0.18	18.452	-0.319	58140000		
	0.454	18.452	-0.319	18800000		
	0.667	18.452	-0.319	64940000		
	1.138	18.452	-0.319	46300000		
	1.31	18.452	-0.319	39220000		
	4.483	18.452	-0.319	13270000		
	5.347	18.452	-0.32	6747000		
	6.384	18.452	-0.32	10560000		
	6.599	18.452	-0.319	9000000		
	6.738	18.452	-0.319	7198000		
	7.889	18.452	-0.318	7031000		
	8.349	18.452	-0.32	23840000		
	8.8	18.452	-0.32	8361000		
	0.926	18.453	-0.317	7111000		
	1.863	18.457	-0.316	6183000		
	2.098	18.456	-0.317	5363000		
	3.67	18.458	-0.321	4527000		
	0.286	18.454	-0.318	10510000		
V14	0.181	21.406	0.183	695500000	V	16.2
	0.649	21.406	0.183	69110000		
	1.139	21.406	0.183	51020000		
	1.292	21.396	0.185	10960000		
	1.656	21.396	0.185	7906000		
	1.864	21.396	0.185	7267000		
	2.975	21.396	0.182	7023000		
	3.282	21.396	0.183	7707000		

	3.679	21.396	0.183	19050000		
	3.75	21.396	0.185	12990000		
	3.899	21.396	0.184	10550000		
	4.482	21.396	0.184	36820000		
	5.025	21.396	0.185	14050000		
	6.383	21.406	0.181	7498000		
	6.596	21.406	0.183	13770000		
	7.887	21.406	0.183	26190000		
	8.353	21.406	0.182	6820000		
	9.336	21.406	0.184	12340000		
	-0.322	21.406	0.183	61150000		
V11	0.304	19.65	0.306	919700000	V	22.1
	0.509	19.65	0.306	96430000		
	1.587	19.65	0.306	68540000		
	2.053	19.65	0.306	36850000		
	2.455	19.65	0.307	9244000		
	4.573	19.65	0.306	9386000		
	4.794	19.65	0.306	17400000		
	5.822	19.65	0.305	14640000		
	6.674	19.65	0.305	13570000		
	6.873	19.65	0.305	13940000		
	7.171	19.649	0.303	8983000		
	8.281	19.66	0.304	9537000		
	8.494	19.647	0.306	14330000		
	0.848	19.65	0.306	10800000		
V10	0.3	21.23	0.505	61740000	V	22.2
	0.504	21.23	0.505	580400000		
	0.846	21.23	0.505	31960000		
	1.589	21.23	0.505	53880000		
	1.7	21.23	0.505	14170000		
	1.908	21.218	0.503	13870000		
	2.054	21.23	0.505	35920000		
	2.134	21.23	0.504	14280000		
	2.46	21.23	0.505	16010000		
	4.586	21.23	0.504	11240000		
	5.822	21.218	0.504	7159000		
	6.67	21.241	0.505	8077000		
	6.877	21.204	0.506	9432000		
	7.176	21.217	0.505	9168000		
	8.272	21.237	0.503	7876000		
	8.492	21.215	0.505	27710000		
V20	0.643	22.037	0.646	716900000	V	26.1
	-0.319	22.037	0.644	15020000		
	0.179	22.037	0.646	36110000		

	0.874	22.055	0.647	12250000		
	0.948	22.048	0.645	12720000		
	1.13	22.025	0.646	39360000		
	1.655	22.023	0.646	54960000		
	1.842	22.026	0.646	19530000		
	2.122	22.036	0.645	25200000		
	2.969	22.022	0.647	7159000		
	3.678	22.022	0.646	18990000		
	4.231	22.022	0.646	29300000		
	4.481	22.022	0.647	9612000		
	5.292	22.018	0.644	5668000		
	6.594	22.024	0.646	15950000		
	8.158	22.018	0.645	19030000		
	9.366	22.028	0.645	18420000		
V21	0.627	21.673	0.626	732400000	V	26.2
	-0.318	21.673	0.625	14780000		
	0.18	21.673	0.626	16200000		
	0.941	21.668	0.626	30520000		
	1.128	21.668	0.626	29060000		
	1.657	21.668	0.626	45880000		
	1.838	21.67	0.624	10380000		
	2.125	21.675	0.626	14660000		
	3.683	21.676	0.626	8292000		
	4.233	21.676	0.626	25370000		
	5.283	21.668	0.626	18210000		
	4.476	21.676	0.627	6014000		
	6.593	21.668	0.627	16010000		
	8.158	21.672	0.626	15290000		
	8.458	21.645	0.627	7076000		
	9.375	21.67	0.626	19010000		
	2.062	21.675	0.627	9378000		
	7.959	21.646	0.625	5597000		
	6.381	21.682	0.63	5396000		
V13	0.62	21.418	0.944	92940000	V	44.1
	0.94	21.418	0.944	436000000		
	1.703	21.418	0.944	40760000		
	2.082	21.418	0.944	15060000		
	2.968	21.429	0.941	8901000		
	3.09	21.406	0.946	6125000		
	3.468	21.418	0.942	12120000		
	3.839	21.418	0.945	11720000		
	4.066	21.418	0.944	7458000		
	5.286	21.418	0.944	24760000		
	6.383	21.418	0.943	30260000		
	6.596	21.418	0.943	26170000		

	6.747	21.406	0.947	7679000		
	7.198	21.398	0.945	6271000		
	7.998	21.419	0.942	16330000		
	8.466	21.439	0.944	7239000		
	1.285	21.454	0.944	8692000		
V12	0.602	20.849	0.602	465400000	V	44.2
	0.939	20.849	0.602	54510000		
	1.71	20.849	0.602	40860000		
	1.843	20.849	0.601	25030000		
	2.133	20.853	0.6	11230000		
	2.024	20.853	0.601	6045000		
	2.32	20.838	0.6	9951000		
	2.434	20.838	0.602	8929000		
	3.433	20.844	0.602	27500000		
	3.833	20.855	0.602	21950000		
	4.792	20.842	0.602	8015000		
	4.882	20.842	0.601	10700000		
	5.285	20.842	0.602	27190000		
	7.988	20.86	0.601	6109000		
	8.173	20.838	0.602	12900000		
	8.465	20.833	0.602	19580000		
	9.342	20.868	0.6	8587000		
V23	-0.32	21.161	0.666	14560000	V	51.1
	0.663	21.167	0.665	605800000		
	0.937	21.167	0.666	26130000		
	1.086	21.165	0.667	6007000		
	1.134	21.16	0.668	4132000		
	1.255	21.165	0.667	6913000		
	1.328	21.149	0.664	5076000		
	1.658	21.167	0.666	12930000		
	1.841	21.154	0.664	10380000		
	2.078	21.167	0.666	41110000		
	3.438	21.166	0.666	28930000		
	3.813	21.199	0.667	6257000		
	4.037	21.167	0.666	18360000		
	5.34	21.148	0.666	7055000		
	6.375	21.15	0.665	6365000		
	7.944	21.162	0.664	16070000		
	8.093	21.168	0.664	26360000		
	9.332	21.168	0.665	20410000		
	9.513	21.204	0.666	7461000		
V26	-0.324	22.279	0.71	33210000	V	51.2
	0.188	22.283	0.71	7310000		
	0.703	22.283	0.71	609700000		

	0.937	22.279	0.711	27980000		
	1.128	22.277	0.71	12550000		
	1.308	22.272	0.71	15480000		
	1.663	22.274	0.711	6170000		
	2.074	22.284	0.71	43910000		
	2.97	22.288	0.709	5021000		
	3.084	22.271	0.71	5293000		
	3.439	22.277	0.713	5088000		
	4.038	22.279	0.71	30770000		
	5.345	22.271	0.711	13990000		
	6.388	22.277	0.71	12950000		
	6.606	22.272	0.711	5023000		
	6.74	22.269	0.71	6405000		
	8.093	22.274	0.71	25480000		
	8.338	22.3	0.711	8649000		
	9.513	22.284	0.711	14320000		
V15	0.324	22.602	0.621	35680000	V	95.1
	0.62	22.61	0.621	450300000		
	0.759	22.61	0.621	50030000		
	0.837	22.61	0.621	25810000		
	0.999	22.61	0.621	55670000		
	1.225	22.61	0.621	18680000		
	1.628	22.61	0.621	13760000		
	1.79	22.61	0.621	37720000		
	4.076	22.597	0.623	8823000		
	4.422	22.594	0.62	24160000		
	4.803	22.594	0.618	10850000		
	7.386	22.594	0.62	7711000		
	8.171	22.583	0.621	6066000		
	9.019	22.591	0.622	11830000		
V17	-0.132	23.639	1.005	6479000	V	95.2
	0.325	23.642	1.005	31630000		
	0.62	23.642	1.005	56290000		
	1.002	23.642	1.005	330000000		
	1.788	23.642	1.005	34930000		
	4.103	23.642	1.005	7004000		
	4.418	23.642	1.005	21740000		
	4.823	23.642	1.005	20860000		
	7.124	23.643	1.001	9167000		
	7.384	23.64	1.005	44000000		
	7.853	23.61	1.005	7097000		
V22	-0.127	22.079	0.861	17280000	V	107.1
	0.326	22.081	0.859	25650000		
	0.619	22.081	0.859	80550000		

	0.86	22.081	0.86	702300000		
	1.258	22.076	0.858	7264000		
	1.453	22.081	0.859	6214000		
	1.716	22.081	0.859	22750000		
	1.881	22.081	0.857	16200000		
	2.054	22.081	0.859	56900000		
	3.092	22.081	0.857	9055000		
	3.429	22.067	0.859	16920000		
	4.045	22.081	0.859	9535000		
	4.793	22.081	0.859	19880000		
	5.247	22.081	0.858	6829000		
	5.725	22.08	0.861	9294000		
	6.724	22.084	0.863	7472000		
	8.834	22.082	0.858	8202000		
	9.407	22.083	0.86	14530000		
	2.258	22.078	0.855	6480000		
V27	-0.128	21.993	0.946	11010000	V	107.2
	0.331	21.993	0.945	13310000		
	0.62	21.993	0.946	48990000		
	0.941	21.993	0.946	685700000		
	1.452	22.019	0.947	9733000		
	1.711	21.991	0.945	24970000		
	1.862	21.978	0.945	23510000		
	2.054	21.993	0.946	45660000		
	2.166	22.011	0.944	9430000		
	3.088	21.992	0.948	7955000		
	3.425	21.993	0.945	19640000		
	4.057	21.99	0.945	12810000		
	4.652	21.993	0.945	5303000		
	4.795	21.993	0.945	21560000		
	5.716	21.992	0.944	9073000		
	6.732	21.988	0.943	6178000		
	8.481	22.004	0.944	5236000		
	8.823	21.979	0.946	12370000		
	8.967	22.005	0.944	5630000		
	9.403	21.985	0.946	6916000		
V25	0.874	22.151	0.881	895600000	V	113.1
	0.997	22.151	0.881	117400000		
	2.132	22.151	0.88	65030000		
	4.014	22.151	0.881	48970000		
	7.554	22.151	0.881	26870000		
	8.47	22.151	0.879	12260000		
V24	0.987	21.442	0.991	1307000000	V	113.2
	1.852	21.442	0.991	8465000		

	2.133	21.442	0.991	78580000		
	2.895	21.442	0.991	9352000		
	3.168	21.442	0.991	7109000		
	4.014	21.442	0.991	46910000		
	7.563	21.442	0.991	11410000		
	8.476	21.442	0.991	29760000		
V18	0.997	21.526	0.999	1038000000	V	123.1
	0.309	21.514	0.998	8105000		
	1.405	21.526	0.998	48490000		
	1.736	21.514	0.996	9872000		
	2.242	21.526	0.998	60100000		
	4.621	21.523	1	18790000		
	4.88	21.523	0.999	34590000		
	5.435	21.523	1.002	6707000		
	6.458	21.526	0.998	5369000		
	6.834	21.526	0.997	4661000		
	7.484	21.526	0.998	5272000		
	7.643	21.526	0.996	6642000		
	8.546	21.526	0.999	26640000		
	9.31	21.525	0.998	12860000		
V19	-0.159	20.393	0.942	6597000	V	123.2
	0.317	20.394	0.945	28560000		
	1.37	20.385	0.948	11730000		
	1.548	20.394	0.948	11850000		
	1.739	20.395	0.945	17450000		
	2.242	20.395	0.946	47150000		
	2.83	20.395	0.946	14410000		
	3.233	20.392	0.943	8706000		
	4.879	20.393	0.945	10310000		
	5.337	20.381	0.948	5386000		
	6.844	20.395	0.948	18560000		
	7.498	20.395	0.946	15820000		
	8.196	20.398	0.945	7908000		
	0.943	20.398	0.945	969500000		
